# Cannabidiol and other non-psychotropic cannabinoids from *Cannabis sativa* as therapeutics for microglial-mediated neuroinflammation and neurodegeneration

**DOI:** 10.1186/s42238-026-00445-5

**Published:** 2026-05-13

**Authors:** Daniel K. Fowler, Tylor M. Savage, Duncan I. Mackie

**Affiliations:** 1 MedPharm Holdings LLC, dba Bud & Mary’s, Denver, CO USA; 2 Department of Pharmaceutical Sciences, Skaggs School of Pharmacy and Pharmaceutical Sciences and The Graduate School, University of Colorado Anschutz Medical Campus, Aurora, CO 80045 USA; 3 Present Address: S3 Genetics, Denver, CO USA

**Keywords:** Cannabinoid, Cannabidiol, Cannabigerol, Cannabichromene, Microglia, Inflammation, Alzheimer’s disease, Parkinson’s disease, Multiple sclerosis, Huntington’s disease

## Abstract

Non-psychotropic phytocannabinoids produced by *Cannabis sativa*, including cannabidiol, cannabigerol, cannabichromene and their varin and acidic analogs, are emerging as promising modulators of neuroinflammation, particularly through actions on microglia, the brain’s resident immune cells. These compounds engage numerous receptors, ion channels, and intracellular signaling systems in microglia associated with neuroinflammation, and therefore are promising therapeutic candidates to treat chronic microglial inflammation-mediated neurodegenerative disorders. Despite substantial public and scientific interest, comprehensive evaluation of their mechanistic diversity, disease-relevant potential, and translational gaps across neurodegenerative disorders remains limited. Commonly, gaps also exist between cannabis breeders’ and cultivators’ knowledge of phytocannabinoid diversity and translational scientists’ understanding of therapeutic potential. In this review, we first provide an in-depth overview of the main non-psychotropic phytocannabinoids, their biosynthesis, and the genetics that control their production in cannabis. We then summarize the known mechanisms of action for each cannabinoid in microglial-expressed molecular targets and signaling pathways relevant to neuroinflammation. Lastly, we review the effects of non-psychotropic phytocannabinoids in pre-clinical models and clinical trials of four neuroinflammation-associated neurodegenerative diseases: Alzheimer’s disease, Parkinson’s disease, multiple sclerosis, and Huntington’s disease. Current evidence supports meaningful biological activity and complex cannabinoid-specific polypharmacology, yet substantial gaps persist, especially for cannabinoids other than cannabidiol; addressing these gaps in disease-relevant models will be essential for translating these compounds into future therapeutic strategies. Further, we anticipate the summarized information will foster collaboration between cannabis breeders/cultivators and applications scientists for therapeutic evaluation and development of emerging non-psychotropic phytocannabinoids.

## Background

### Overview of the endocannabinoid system (ECS) and its role in neuroinflammation and neurodegeneration

Inflammation is a coordinated cellular response in which immune cells release signaling molecules such as cytokines, chemokines, and reactive mediators that amplify, sustain, and eventually resolve tissue injury-associated stress signals (Medzhitov 2008). Neuroinflammation represents this process within the central nervous system (CNS), where resident microglia, infiltrating peripheral macrophages, and reactive astrocytes release pro-inflammatory cytokines, chemokines, and reactive oxygen/nitrogen species (ROS/RNS) in response to protein aggregates, neuronal injury signals, or pathogens (Glass et al., 2010). Chronic neuroinflammation drives neurodegeneration by creating a self-perpetuating cycle where inflammation impairs synaptic function, accelerates protein aggregation, and directly induces neuronal apoptosis (Ransohoff 2016).

The endocannabinoid system (ECS) is a conserved lipid signaling network that maintains homeostasis across the CNS, immune system, and peripheral organs. The core ECS consists of endogenous lipid signaling ligands, primarily anandamide (AEA) and 2‑arachidonoylglycerol (2-AG), their canonical G protein-coupled receptors (GPCRs), cannabinoid receptor 1 (CB1) and cannabinoid receptor 2 (CB2), and the enzymes responsible for ligand synthesis and degradation (Lu and Mackie 2021). CB1 is highly expressed in neurons throughout the CNS, where it modulates neurotransmitter release, synaptic plasticity, and neuronal excitability (Mackie 2006). In contrast, CB2 is enriched in immune cells, including macrophages, microglia, B cells, T cells, and other leukocytes, and is strongly inducible under inflammatory conditions (Atwood and Mackie 2010). This differential expression pattern underlies the functional specialization of CB1 and CB2 in neural versus immune regulation.

Endocannabinoids AEA and 2-AG are synthesized on demand from membrane phospholipid precursors rather than stored in vesicles, via enzymes N-acyl phosphatidylethanolamine-specific phospholipase D (NAPE-PLD) and diacylglycerol lipases (DAGLα/β), respectively (Piomelli 2003). AEA is primarily degraded by fatty acid amide hydrolase (FAAH) (Cravatt et al., 1996), while 2-AG is hydrolyzed by monoacylglycerol lipase (MAGL) (Dinh et al., 2002). Tight spatiotemporal regulation of endocannabinoid synthesis and degradation allows the ECS to function as a local feedback system that limits excessive cellular activation. In immune cells, this feedback loop constrains pro-inflammatory signaling, cytokine release, oxidative stress, and cell migration (Pandey et al., 2009).

Beyond CB1 and CB2, the ECS encompasses a broader signaling network that includes transient receptor potential (TRP) channels, peroxisome proliferator-activated receptors (PPARs), other GPCRs such as GPR55, and enzymes involved in lipid metabolism (Di Marzo and Piscitelli 2015). Many endocannabinoids and phytocannabinoids produced by the plant *Cannabis sativa* L. interact with these non-canonical targets, contributing to CB1 and CB2-independent anti-inflammatory and protective effects. Within the CNS, the ECS plays a particularly important role in regulating neuroinflammation, primarily through microglia (Stella 2009), the resident immune cells of the brain, but also through astrocytes and infiltrating peripheral immune cells, and neuron-microglia crosstalk (Stella 2010). Cannabidiol (CBD) and several other non-psychotropic cannabis phytocannabinoids have been shown to reduce neuroinflammation, and have attracted interest as potential therapeutics for neuroinflammation-associated neurodegenerative diseases including Alzheimer’s disease (AD), Parkinson’s disease (PD), multiple sclerosis (MS), Huntington’s disease (HD), and amyotrophic lateral sclerosis (ALS) (Cristino et al., 2020).

### Definition and classification of non-psychotropic phytocannabinoids

We define non-psychotropic cannabinoids as compounds that interact with the body's ECS but do not produce the consciousness-altering “high” associated with Δ9-tetrahydrocannabinol (THC). We purposely avoid 'intoxicating' as it implies alcohol-like inebriation, and does not accurately represent the psychoactive properties of THC, which is commonly not associated with alcohol-like functional impairment, especially during chronic medical cannabis treatment (Sagar et al., 2021; Eadie et al., 2021; Wieghorst et al., 2022). THC produces psychotropic effects through its high-affinity, low-dose agonism of CB1 (Pertwee 2008). Non-psychotropic cannabinoids are therefore defined as those lacking significant low-dose CB1 agonist activity. Collectively and as explained in more detail later in this review, non-psychotropic phytocannabinoids are all cannabis-produced cannabinoids other than THC itself.

### Scope and structure of the review

The scope and intent of the review is to 1) provide an overview of all known non-psychotropic cannabinoids biosynthesized by cannabis and the genetics that control their production, 2) summarize the known mechanisms of action (MOA) for each non-psychotropic cannabinoid in microglial-expressed molecular targets and 3) discuss the therapeutic potential of non-psychotropic phytocannabinoids in microglial-mediated neuroinflammation and what is known about their action in four neurodegenerative diseases: AD, PD, MS, and HD.

## Main text

### Non-psychotropic cannabinoids produced in cannabis

**Fig. 1:**
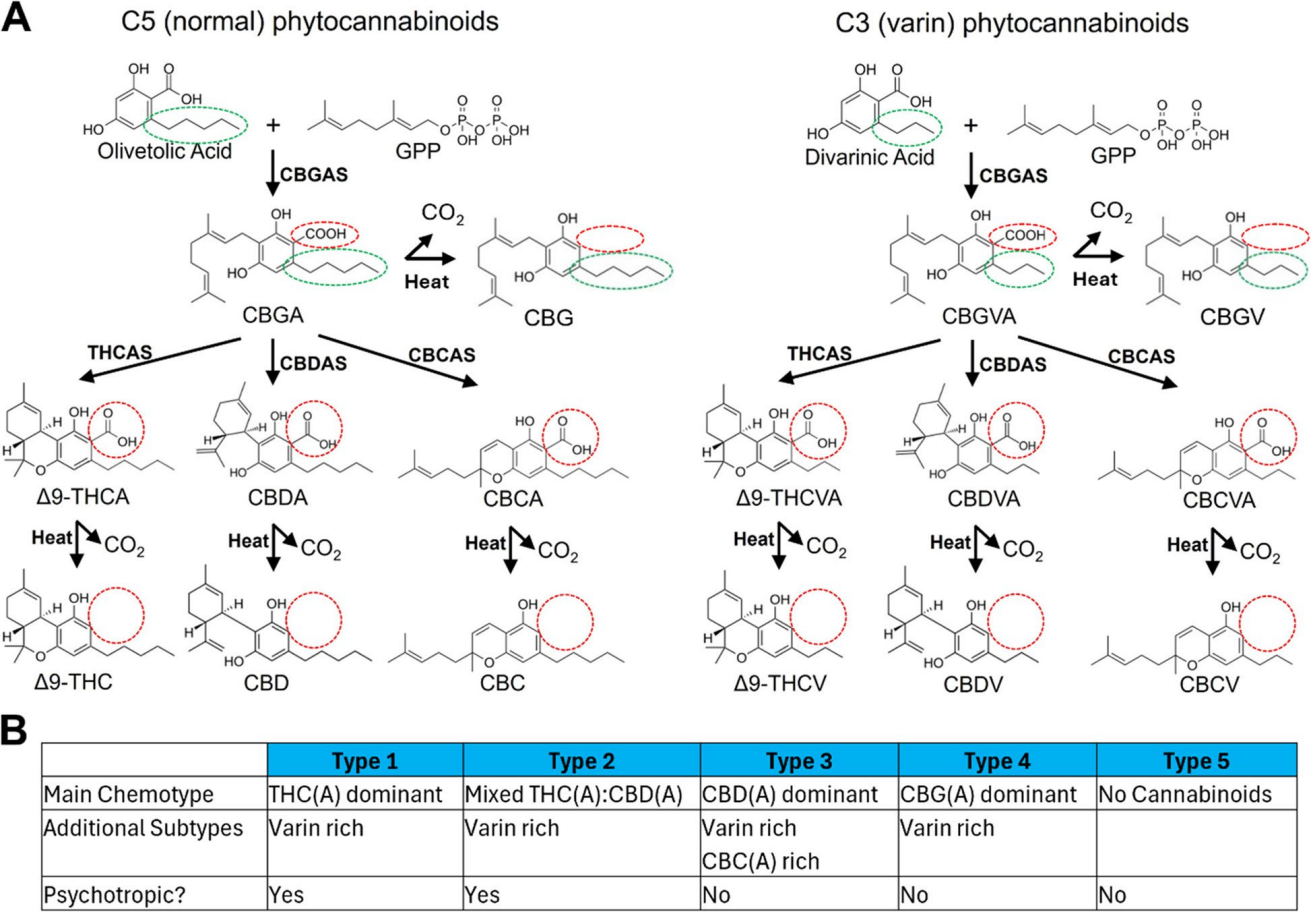
Cannabis cannabinoids and plant chemical phenotypes (chemotypes). **A** Biosynthesis and decarboxylation of normal (5-carbon alkyl tail) and varin (3-carbon alkyl tail) cannabinoids. Alkyl tail length (green circles) is maintained throughout synthesis and heat-mediated decarboxylation (red circles). **B** Main chemotypes of cannabis plants, additional cannabinoid subtypes, and associated psychotropic potential

Cannabinoid biosynthesis pathways in cannabis are presented in Fig. 1A. Cannabis biosynthesizes acidic cannabinoids cannabigerolic acid (CBGA), Δ9-tetrahydrocannabinolic acid (THCA), cannabidiolic acid (CBDA), cannabichromenic acid (CBCA) and their 3-carbon alkyl tail “varin” counterparts: cannabigerovarinic acid (CBGVA), Δ9-tetrahydrocannabivarinic acid (THCVA), cannabidivarinic acid (CBDVA), and cannabichromevarinic acid (CBCVA). In all cases, heat causes decarboxylation of acidic cannabinoids into their neutral forms, e.g. CBDA to CBD (Fig. 1A, red circles). Total cannabinoid content of an acidic and neutral species is often discussed and is denoted as a parenthesized (A) following the cannabinoid acronym, e.g. CBD(A) for total CBDA and CBD content. The full list of non-psychotropic cannabis phytocannabinoids is as follows: CBD, cannabidivarin (CBDV), CBDA, CBDVA, cannabigerol (CBG), cannabigerovarin (CBGV), CBGA, CBGVA, cannabichromene (CBC), cannabichromevarin (CBCV), CBCA, CBCVA, THCA, Δ9-tetrahydrocannabivarin (THCV), and THCVA. Importantly, although THCV has been reported to be psychotropic in humans at very high doses (> 100 mg) (Englund,et al., 2016), we consider it to be non-psychotropic for the purposes of this review due to its lack of low-dose agonism at CB1 (Walsh and Holmes 2022). Because THCA readily decarboxylates into THC, it is considered a potential psychotropic, although THCA itself is non-psychotropic.

Classification and scientific understanding of the genetics that control major and minor cannabis chemical phenotypes (chemotypes) is important for breeding and utilization of the plant across its natural spectrum of biologically active and potentially therapeutic cannabinoid content. Selective breeding has led to cannabis cultivars which are grown for cannabinoids as their major metabolite across their chemotype spectrum. This review considers the main cannabinoids produced by cannabis in appreciable amounts, typically > 1% by weight of dry flower weight (w/w), across the current known diversity of cannabis genotypes.

In most cannabis varieties, THCA synthase (THCAS) and/or CBDA synthase (CBDAS) enzymatically cyclize precursor CBGA into THCA and CBDA, respectively (Taura et al., 1995; Taura et al., 2007) (Fig. 1A). Cannabis plants currently present as five major chemotypes as follows: THCA-dominant (Type 1), mixed THCA and CBDA (Type 2), CBDA-dominant (Type 3), CBGA-dominant (Type 4) and no cannabinoids (Type 5) (Fig. 1B). Because of the significant portion of THC(A) produced in Type 1 and 2 chemotypes, these are psychotropic and commonly considered ‘marijuana’ varieties. Type 3—5 plants are non-psychotropic due to the low quantity or absence of THC(A) and are commonly considered ‘hemp’ varieties. Currently, investigational and translational scientists and regulators may be unaware of advances in cannabinoid breeding and the diversity of cannabis phytocannabinoids. Therefore, we review cannabis genetics that control major and minor cannabinoid chemotypes.

#### CBDA and THCA: the major terminal cannabinoids

Recent genomic investigations have shown that THCAS and CBDAS exist in co-dominant, non-recombining alleles which dictate major cannabinoid chemotype (Lynch et al., 2025). Because cannabis is a diploid organism, plants with two THCAS alleles are THC(A)-dominant (Type1), plants with two CBDAS alleles are CBD(A)-dominant (Type 3), and plants with one allele of each are THC(A)- and CBD(A)-codominant (Type 2). The variable THC(A):CBD(A) ratio in Type 2 varieties may arise from allelic variations such as copy number differences (Vergara et al., 2019). It is notable that a major cannabinoid synthase allele with copies of both functional CBDAS and THCAS has not been described.

Type 1 varieties have been selectively bred for high THC(A) content over the last several decades and can produce > 30% THC(A) (w/w), although most commercial cultivators produce flower in the teens-to-twenties percent THC(A) (Clarke and Merlin 2016; Jikomes and Zoorob 2018). Type 2 cultivars have been bred to produce over 20% CBD(A) (w/w) in recent years by introgression of CBDAS into high-cannabinoid capacity marijuana varieties (Grassa et al., 2021). High levels of cannabinoids in current cannabis material are a marked increase from 1—3% observed THC(A) and CBD(A) in the 1970 s (Small and Beckstead 1973) and an average ~ 4% THC(A) found in illicit cannabis seized by the U.S. Drug Enforcement Administration in 1995 (ElSohly et al., 2016).

Type 1 plants, and to a much less extent Type 2 plants, make up the vast majority of chemotypes cultivated for medical and recreational cannabis markets in the US (Smith et al., 2022; Pennypacker et al., 2022), and likely other jurisdictions. Of the first three chemotypes, only Type 3 varieties can be considered non-psychotropic. However, currently all known CBDAS variants non-specifically produce THCA in a fixed ratio of ~ 20:1 CBDA:THCA (Zirpel et al., 2018). Therefore, a CBD(A)-dominant plant with 20% CBD(A) (w/w) is expected to contain ~ 1% THC(A) (w/w).

#### CBGA: the central biosynthetic precursor of phytocannabinoids

CBGA is the first cannabinoid produced in the cannabis biosynthetic pathway, through condensation of geranyldiphosphate (GPP) and olivetolic acid by the aromatic prenyltransferase CBGA synthase (CBGAS) (Fellermeier and Zenk 1998) (Fig. 1A). Most cannabis genotypes will further convert CBGA into THCA and/or CBDA, but these genotypes will still contain some fraction of unconverted CBGA, typically less than 1% (w/w) (Coogan 2019). This residual CBG(A) content can be significant in cannabis concentrates of Type 1—3 plants, at single digit percents (w/w) (Giordano et al., 2025).

Recent genomic studies have shown Type 4 chemotypes stem from nonfunctional mutations in either THCAS or CBDAS leading to precursor CBGA accumulation, and hypofunctional mutations can produce plants with spectrums of CBG(A):THC(A) and CBG(A):CBD(A) ratios (Onofri et al., 2015; Garfinkel et al., 2021). Type 4 plants have been similarly bred for cannabinoid content in recent years, with current cultivars able to produce over 10% CBG(A) (w/w) (Crawford et al., 2021).

#### CBCA: the least-understood terminal cannabinoid

A dedicated CBCA synthase (CBCAS) enzyme cyclizes CBGA to CBCA in cannabis (Morimoto et al., 1998) (Fig. 1A). Sequences of CBCAS were only recently discovered in the cannabis genome, and selectively produced CBCA when expressed in yeast fed with CBGA (Laverty et al., 2019). Despite these advances, the genetics underlying CBCA production by cannabis remain unclear. Several studies have shown a large copy number diversity of CBCAS (Grassa et al., 2021; Fulvio et al., 2021), with the most recent cannabis pangenome analysis showing CBCAS in 56% of cannabis genomes in arrays of 1—15 copies, spaced apart from THCAS and CBDAS cassettes on the same chromosome (Lynch et al., 2025). However, CBCAS expression was low across all tissues and cultivars (Lynch et al., 2025; Fulvio et al., 2021).

CBC(A) concentration in cannabis flower rarely exceeds trace amounts and is considered a minor cannabinoid. Indeed, an analysis of over 80,000 commercial cannabis samples across the U.S. showed CBC(A) was virtually non-existent in Type 1 cannabis (Smith et al., 2022). In contrast, recent screening of Type 3 hemp cultivars found varieties with CBC(A) concentrations up to 4% (w/w) (Torres et al., 2026), suggesting a novel CBC(A)-rich cannabis subtype with commercial and therapeutic implications (Fig. 1B).

#### Varinic phytocannabinoids: structural and functional distinctions

Cannabinoids that are structurally homologous but contain a three-carbon alkyl tail rather than a five-carbon tail are collectively termed “varin” cannabinoids. Cannabis produces varinic cannabinoids via condensation of GPP with propyl (C3) side chain precursor divarinic acid, yielding CBGVA instead of pentyl (C5) side chain precursor olivetolic acid by CBGAS (Tahir et al., 2021). Cannabinoid synthases are promiscuous for substrate and cyclize CBGVA into THCVA, CBDVA, and CBCVA (Luo et al., 2019) (Fig. 1A).

Contemporary studies have described cultivars with a varinic cannabinoid fraction up to 69% or more, and proposed varin-rich chemotypes are controlled by non-cannabinoid synthase genetic loci (De Meijer and Hammond 2016; Welling et al., 2018). Alleles favoring upstream carbon flux of precursors into C3 instead of C5 substrates are hypothesized to set varin cannabinoid ratio, and a recent pan-genome analysis showed variation in acyl-lipid thioesterase genes were linked with varin proportion (Lynch et al., 2025). Because alkyl chain length is determined biosynthetically prior to CBGAS prenylation (Fig. 1A, green circles), all cannabinoid-producing cannabis types (Types 1—4) can contain varin-rich subtypes (Fig. 1B).

#### Non-botanical, semi-synthetic and synthetic derivatives

Cannabinoids can be converted to other cyclic forms or modified structurally in the lab. These are commonly referred to as “semi-synthetic” cannabinoids. We do not recognize cannabinoids with atypical cyclic structures (e.g. Δ8- and Δ10-THC) or hydrogenation patterns (e.g. hexahydrocannabinol (HHC) and hexahydrocannabidiol (H4CBD)) as botanically produced cannabinoids and are therefore out of scope of this review.

Further, although cannabinoids with atypical alkyl chain lengths (e.g. other than 3 or 5 carbons) have been detected in trace amounts, we consider cannabinoids with abnormal alkyl chains as likely of synthetic origin. For example, to the best of our knowledge the heptyl (C7) chain Δ9-tetrahydrocannabiphorol (THCP) has only been reported at trace concentrations in botanical cannabis samples ranging from 0.0023% to 0.0136% (w/w) (Citti et al., 2019; Bueno and Greenbaum 2021). Due to the impracticality of purifying THCP from these concentrations, and because THCP found in recreational products is commonly found with other synthetic and semi-synthetic cannabinoids, THCP is hypothesized to be synthetically produced and not botanically extracted (Caprari et al., 2024).

Most *in vitro*, *in vivo*, and clinical research on non-psychotropic phytocannabinoids reviewed here utilize botanically-derived and purified cannabinoids. Studies that utilized synthetically produced cannabinoids are indicated where appropriate.

### Non-psychotropic phytocannabinoid MOAs in microglial-mediated neuroinflammation

#### The role of microglia in neuroinflammation

Across the inflammatory neurodegenerative disease spectrum, microglia act as the primary source of both pro- and anti-inflammatory signals (Colonna and Butovsky 2017). Non-pathologically activated microglia, often referred to as M0 phenotype, constantly survey their surroundings and transition to reactive states when they encounter triggers like protein aggregates (Nimmerjahn et al., 2005). Experimentally, lipopolysaccharide (LPS), a major glycolipid component of the outer membrane of Gram-negative bacteria, is very commonly used to activate microglia via toll-like receptor 4 (TLR4) signaling, although its ability to model CNS injury beyond neural infection is debated (Lehnardt et al., 2003). Activated microglia, often referred to M1 phenotype, secrete canonical pro-inflammatory cytokines including tumor necrosis factor-alpha (TNF-α), interleukin-1 beta (IL-1β), interleukin-6 (IL-6), and interleukin-12/interleukin-23 (IL-12p70, IL-23), which can be directly neurotoxic (Smith et al., 2012). Pro-inflammatory microglia also release chemokines such as C–C motif chemokine ligands 2, 3, and 4 (CCL2, 3, 4) and C-X-C motif chemokine ligands 8 and 10 (CXCL-8, −10) to attract further microglia and sometimes peripheral immune cells to injury sites (Ransohoff 2009). In contrast, alternatively-activated anti-inflammatory microglia, often referred to as M2 phenotype, promote tissue repair and resolution by secreting canonical anti-inflammatory cytokines such as interleukin-4 (IL-4), interleukin-10 (IL-10), and transforming growth factor-beta (TGF-β) (Colton 2009). Anti-inflammatory microglia also express phagocytic receptors CD206 and arginase-1 (Arg-1) that enhance clearance of protein aggregates and cellular debris while producing growth factors brain-derived neurotrophic factor (BDNF), insulin-like growth factor 1 (IGF-1) and glial cell line-derived neurotrophic factor (GDNF) that support neuronal survival, synaptic remodeling, and neuroprotection (Cherry et al., 2014). It is important to note the M0/M1/M2 nomenclature is an oversimplification of a vast spectrum of microglial heterogeneity, and to emphasize the complex capacity of microglia to either exacerbate or resolve neuroinflammation across neurodegenerative conditions (Ransohoff 2016).

**Fig. 2:**
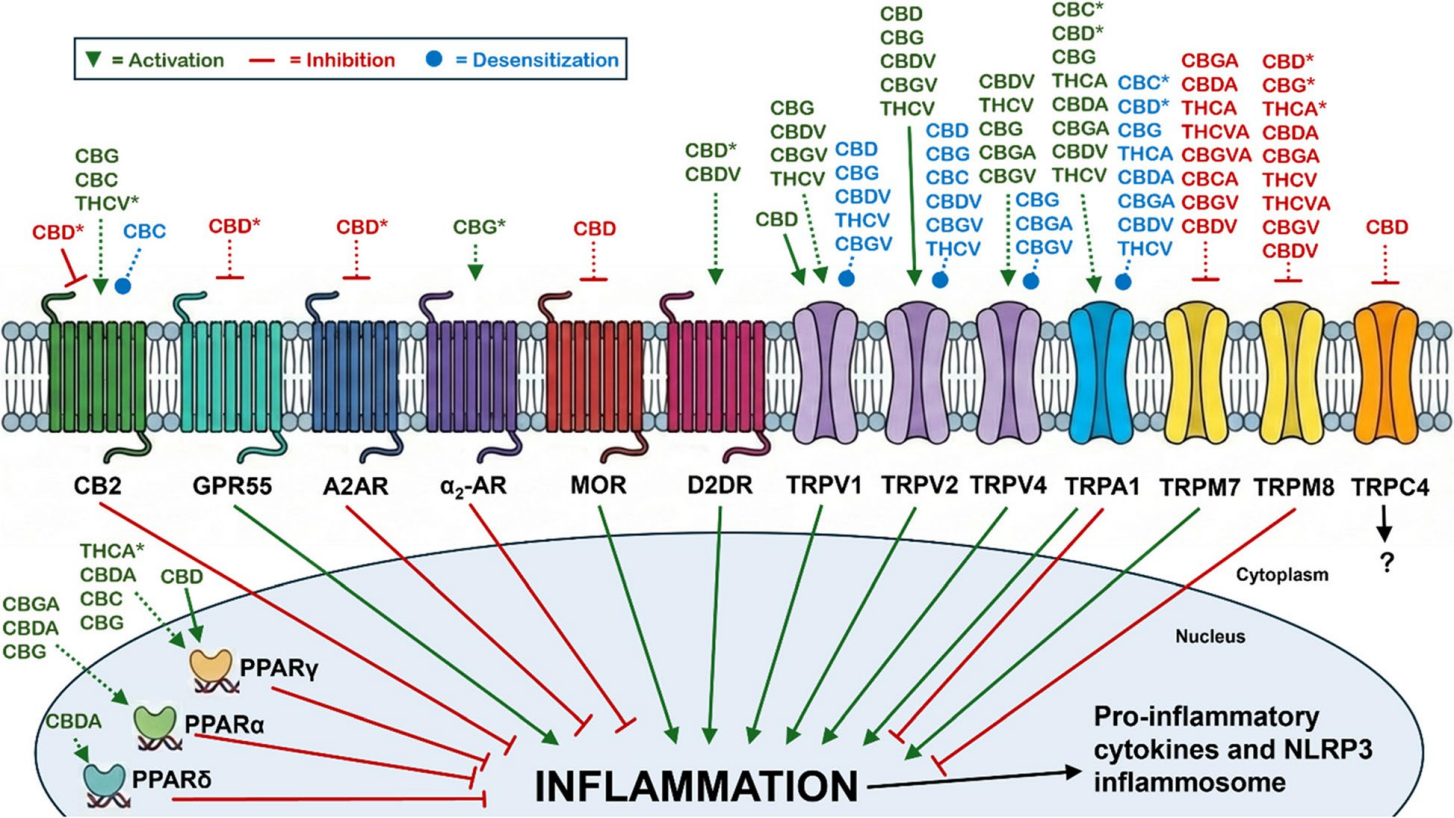
Non-psychotropic phytocannabinoid modulation of GPCR-, TRP channel-, and PPAR-mediated inflammatory signaling in microglia. Solid lines indicate effects directly shown in microglia, dotted lines indicate effects inferred from non-microglial cell types, and asterisks indicate functional interactions at nanomolar concentrations (< 1 µM)

Non-psychotropic phytocannabinoids modulate several known molecular targets expressed in microglia. Below, we review microglial GPCRs, TRP channels, and PPARs that interact with non-psychotropic phytocannabinoids and modulate inflammatory signaling. Figure 2 presents a general overview of the known phytocannabinoid-receptor interactions, general pro- and anti-inflammatory receptor signaling, and whether the effects have been directly demonstrated in microglia (solid lines) or inferred from interactions in other cell types (dotted lines), and if the interaction is significant at nanomolar concentrations (asterisks).

#### Common translational limitations of cannabinoid-microglia research

Key translational limitations in evaluating non-psychotropic phytocannabinoids as modulators of microglial function in neurodegenerative disease are the incomplete understanding of their pharmacokinetic (PK) properties, including oral bioavailability, metabolic fate, and the relationship between systemic exposure and concentrations achieved within the central nervous system. Many cannabinoids, particularly CBD and structurally related compounds, display low and highly variable oral bioavailability due to poor aqueous solubility, extensive first-pass metabolism, and substantial plasma protein binding (Ohlsson et al., 1986; Millar et al., 2018; Moazen-Zadeh et al., 2024). As a result, plasma levels attained with commonly used dosing regimens may be substantially lower than the concentrations required to modulate several molecular targets identified *in vitro*, including those implicated in microglial activation pathways, where reported potencies often fall in the micromolar range (Millar et al., 2018; Moazen-Zadeh, et al., 2024; Patel et al., 2021; Perkins et al., 2020). In addition, cannabinoids undergo complex biotransformation into multiple metabolites whose pharmacological activity in microglia and other CNS-resident immune cells remain largely undefined (Millar et al., 2018; Huestis 2007; Zendulka et al., 2016). Furthermore, most *in vitro* neuroinflammation experiments with microglia utilize LPS for inflammation induction, which may be less relevant than neurodegenerative disease-specific inflammatory mediators like protein aggregates in the context of chronic neuroinflammation. These factors complicate direct extrapolation from cell culture systems, particularly immortalized microglial lines like BV-2, to *in vivo* conditions in humans and highlight the importance of integrating PK and pharmacodynamic data when assessing therapeutic potential.

Equally important is whether enough of these compounds reach microglial populations within the brain. Although cannabinoids are highly lipophilic and capable of crossing biological membranes, blood–brain barrier (BBB) permeability varies across individual phytocannabinoids and may be influenced by active efflux transporters, formulation, protein binding, and disease-associated BBB alterations (Millar et al., 2018; Calapai et al., 2020; Grotenhermen 2003). Available evidence indicates that CBD and some minor cannabinoids can enter the brain, but quantitative data on regional distribution, free (unbound) drug concentrations, and exposure specifically within microglial niches are limited (Calapai et al., 2020; Schouten et al., 2024; Sadaka et al., 2021). Moreover, neurodegenerative conditions themselves can modify BBB integrity and microglial phenotypes, potentially altering drug access and responsiveness (Erickson and Banks 2013; Sweeney et al., 2018; Varatharaj and Galea 2017). Emerging delivery approaches, including lipid-based carriers, nanoemulsion formulations, prodrug strategies, and alternative routes of administration may enhance CNS penetration and improve targeting of microglial cells while minimizing systemic exposure (Grifoni et al., 2025; Teleanu et al., 2018). Future studies should therefore prioritize rigorous PK characterization, brain distribution analyses, and direct measures of target engagement in microglia *in vivo* to determine whether the anti-inflammatory and neuroprotective effects observed in experimental models can be achieved at clinically safe and therapeutically relevant doses in humans.

#### GPCRs in endocannabinoid and phytocannabinoid pharmacology and microglial inflammation

##### CB2

CB2 is a G_i/o_-coupled GPCR that suppresses pro-inflammatory microglial responses. Foundational research with CB2 knockout mice demonstrated that CB2, not CB1, mediated anti-inflammatory effects of THC (Buckley et al., 2000). Mechanistically, endocannabinoids 2-AG and AEA restrain microglial activation primarily through CB2-mediated inhibition of pro-inflammatory cytokine secretion, via suppression of the nuclear factor kappa-light-chain-enhancer of activated B cells (NF-κB) signaling and NLR family pyrin domain containing 3 (NLRP3) inflammasome activation (Tanaka et al., 2020). As the master transcriptional regulator of the innate immune response, NF-κB coordinates the expression of a broad array of pro-inflammatory genes, including those encoding for cytokines, chemokines, and inducible enzymes (O'Neill and Kaltschmidt 1997). In contrast, the NLRP3 inflammasome serves as a specialized multiprotein platform that senses cellular stress and damage-associated cues to catalyze the proteolytic maturation and secretion of the highly potent pyrogenic cytokines IL-1β and IL-18 (Martinon et al., 2002). Endocannabinoids also shift microglia toward anti-inflammatory states through CB2 signaling, upregulating IL-10, TGF-β, and Arg-1 alongside enhanced phagocytosis of protein aggregates via CD206 and triggering receptor expressed on myeloid cells 2 (TREM2) signaling (Mecha et al., 2015; Keren-Shaul et al., 2017; Li and Barres 2018. In neuroinflammatory contexts, neurons, astrocytes and activated microglia produce endocannabinoids which act as negative feedback brakes, preventing excessive microglial reactivity, ROS/RNS production, and recruitment of peripheral monocytes while preserving homeostatic surveillance functions (Stella 2009).

CB2 agonists suppress pro-inflammatory microglial phenotypes through several parallel signaling pathways. G_αi_ inhibits adenylyl cyclase (AC) (Walter et al., 2003; Slipetz et al., 1995), lowers cyclic adenosine monophosphate (cAMP) levels to suppress protein kinase A (PKA) activity, which prevents the phosphorylation and subsequent degradation of inhibitor of kappa-B (IκB) (Tao et al., 2016), retaining NF-κB p65/p50 in the cytoplasm and blocking its nuclear translocation. G_βγ_ scaffolds with β-arrestin2, activating a mitogen-activated protein kinase (MAPK) cascade and extracellular signal-regulated kinase 1/2 (ERK1/2) (Franklin et al., 2013), upregulating nuclear receptor Nurr1 which physically associates with NF‑κB to repress inflammatory gene transcription (Han et al., 2022). G_βγ_/β-arrestin2 also transiently upregulates mitogen-activated protein kinase phosphatase-1 (MKP-1), terminating MAPK signaling and reducing IκB phosphorylation/degradation (Eljaschewitsch et al., 2006). G_βγ_ also recruits phosphoinositide 3-kinase (PI3K)/Akt signaling, inhibiting NF‑κB co-activator glycogen synthase kinase 3 beta (GSK3β)(85). CB2 activation similarly restrains NLRP3 inflammasome assembly by promoting ubiquitination/autophagy-mediated NLRP3 clearance via G_βγ_/AMP-activated protein kinase (AMPK)/unc-51 like autophagy activating kinase 1 (ULK1) signaling, preventing caspase-1-mediated maturation of IL-1β and IL-18 (Jiang et al., 2022).

CB2 activation also promotes anti-inflammatory microglia states. CB2 signaling can reduce signal transducer and activator of transcription 1 (STAT1) activation (Ehrhart et al., 2005) while G_βγ_ signaling recruits PI3K/Akt to enhance STAT3 phosphorylation and nuclear translocation, promoting IL-10, TGF-β, and Arg-1 transcription (Han et al., 2022). These multiple signaling tracks ensure suppression of pro-inflammatory cytokines while inducing anti-inflammatory cytokine and phagocytic signaling pathways. Importantly, CB2 exhibits biased signaling, with different agonists preferentially engaging either G_αi_- or β-arrestin2-dependent pathways (Soethoudt et al., 2017).

Amongst the list of non-psychotropic cannabis phytocannabinoids, CBC, CBG, and THCV have been reported as CB2 agonists or partial agonists. THCV was systematically demonstrated as a partial human CB2 agonist at low nanomolar concentrations in heterologous cells (Pertwee 2008; Thomas et al., 2005). Pharmacological blockade of mouse CB2 in carrageenan‑induced paw edema and formalin‑test pain behavior *in vivo* decreased the anti-inflammatory effect of THCV (1—10 mg/kg, i.p.), suggesting at least partial function through CB2 (Bolognini et al., 2010). CBC was first reported as a human CB2 agonist at micromolar concentrations when expressed in heterologous cells, and prolonged activation by CBC desensitized the receptor (Udoh et al., 2019). It was subsequently shown that only the (−)-CBC enantiomer but not (+)-CBC from synthesized CBC was a human CB2 agonist in heterologous cells, with an EC_50_ of 1.5 µM (Udoh et al., 2025). Despite agonist potential at CB2, the anti-inflammatory effect of CBC has not been directly demonstrated to function through CB2. CBG was recently characterized as a weak partial agonist at human CB2 (EC_50_ ~ 1—3 μM) by using live-cell functional assays demonstrating G_i/o_-coupled cAMP inhibition and β-arrestin recruitment in recombinant HEK-293T cells (Navarro et al., 2018). Unlike CBC, the anti-inflammatory effect of CBG has been shown to be mediated by CB2 in mouse RAW264.7 macrophages challenged with hydrogen peroxide: pharmacological blockade of CB2 with selective antagonist AM630 abolished CBG-induced RNS, inducible nitric oxide synthase (iNOS) expression, and NF‑κB nuclear translocation (Giacoppo et al., 2017). Neither CBC, CBG, or THCV have yet been confirmed to function through CB2 via genetic loss-of-function studies, like receptor knockout mouse models.

CBD acts as an antagonist/inverse agonist at human CB2 expressed in heterologous cells with functional antagonism in the nanomolar range (Thomas et al., 2007), which suggests its anti-inflammatory actions are mediated through CB2-independent mechanisms. Intriguingly, however, recent studies show that CBD’s anti-inflammatory effects in microglia are at least partly CB2 dependent. One study used mouse BV-2 microglia and reported that CBD (sub‑micromolar to low micromolar) reduced LPS‑induced IL‑1β and NLRP3 expression and caspase-1 activity; these effects were significantly blunted by AM630 suggesting CBD inhibition of the NLRP3 inflammasome is partially CB2-dependent (da Silva et al., 2024). In another study, CBD treatment (10 mg/kg, i.p.) inhibited post-traumatic stress disorder (PTSD)-related microglial activation and pro-inflammatory changes in mice, which were diminished by a AM630 and abolished in CB2 knockout mice (Xie et al., 2024). This study also showed that CBD dramatically increased hippocampal AEA levels (Xie et al., 2024), providing a possible indirect CB2-dependent mechanism from CBD treatment.

##### CB1

CB1 is expressed in microglia and CNS infiltrating macrophages (Walter et al., 2003; Cabral 2002; Benito et al., 2005). Recent mouse research with microglial-specific CB1 knockout showed decrease in expression of brain IL‑1β, IL-6 and TNF‑α when challenged with LPS (De Meij et al., 2021), suggesting that microglial CB1 signaling promotes the synthesis of these cytokines. However, the role of CB1 in microglial-mediated inflammation is less central than CB2 and often tightly coupled to neuronal CB1 signaling and neuron-microglia crosstalk. Crucially, γ-aminobutyric acid (GABA)ergic neuron-specific deletion of CB1 leads to microglial activation and neuroinflammation, indicating that CB1 signaling is anti-inflammatory indirectly via neuron-microglia crosstalk (Albayram et al., 2011; Ativie et al., 2018). Like CB2, CB1 also displays biased signaling between G_αi_/AC and G_βγ_/β-arrestin pathways depending on agonist (Thorsen et al., 2025).

THCA acts as both a low affinity orthosteric agonist and a positive allosteric modulator of human CB1 expressed in heterologous cells (Palomares et al., 2020). While not directly involving neuroinflammation, several anti-inflammatory effects of THCA (20 mg/kg, i.p.) in collagen-induced arthritis in mice *in vivo*, including reduced expression of iNOS, pro-inflammatory cytokines IL-6, TNF‑α, IL‑1β and IL-17 and chemokine CXCL16, were blocked by the CB1 antagonist SR141716 (Palomares et al., 2020), suggesting at least partial CB1-dependency. Like at CB2, CBD is a low affinity inverse agonist at mouse brain membrane CB1 (Thomas et al., 2007) as well as a negative allosteric modulator of human CB1 expressed in heterologous cells (Laprairie et al., 2015). THCV is an antagonist at low concentrations and partial agonist at high concentrations at human CB1 expressed in heterologous cells (Pertwee 2008; Thomas et al., 2005). CBG is a low affinity partial agonist of CB1, and reduces the effect of AEA at human CB1 in heterologous cells, acting functionally as a low-efficacy antagonist/competitor (Navarro et al., 2018). There is currently no strong pharmacological or genetic data showing CBD, THCV or CBG anti-inflammatory effects are CB1-dependent.

##### G Protein-Coupled Receptor 55 (GPR55)

GPR55 is a GPCR receptor primarily for lysophosphatidylinositol (LPI) (Oka et al., 2007), and is functionally expressed in cultured BV-2 and primary mouse and rat microglia (Pietr et al., 2009; Saliba et al., 2018). Disputed early results showed 2-AG and AEA as potent human GPR55 agonists via a GTPγS assay (Ryberg et al., 2007), and GPR55 was subsequently proposed as the “third” endocannabinoid receptor CB3 (Ross 2009). However, subsequent studies using calcium mobilization assays and β-arrestin translocation assays in human GPR55-expressing HEK-293 cells found either no activation by AEA/2-AG or only very weak/partial responses compared to LPI (Sharir and Abood 2010). Regardless of the endocannabinoid discrepancy, GPR55 is a target of CBD and plays functional roles in microglia inflammation. In primary mouse microglia, GPR55 expression decreases upon activation with LPS or interferon gamma (IFNγ), suggesting a homeostatic role (Pietr et al., 2009). GPR55 antagonists ablated LPS-induced prostaglandin E2 (PGE2) release in primary rat microglia, suggesting GPR55 drives the arachidonic acid (AA) cascade in neuroinflammation independent of CB2 (Saliba et al., 2018). Pharmacological activation of GPR55 in human THP-1-derived macrophages and primary human monocytes and natural killer (NK)-cells upregulates TNF‑α (Lanuti et al., 2015; Chiurchiù et al., 2015), although this has not yet been demonstrated in microglia. GPR55-mediated calcium influx involves G_q_, G_12_, RhoA, actin, phospholipase C (PLC), and calcium release from inositol 1,4,5-trisphosphate receptor (IP_3_R)-gated stores in recombinant cells (Lauckner et al., 2008).

CBD is a confirmed antagonist (IC_50_ ~ 445 nM) of human GPR55 expressed in heterologous cells (Ryberg et al., 2007; Sharir and Abood 2010), and synthetic CBD counteracted enhanced TNF‑α expression induced by GPR55-selective agonist O-1602 in human THP-1-derived macrophages (Lanuti et al., 2015). However, there is no direct evidence using GPR55 genetic loss-of-function showing CBD’s anti-inflammatory properties are GPR55-dependent, or that CBD functions through GPR55 in microglia.

##### Adenosine A_2A_ receptor (A2AR)

A2AR is stimulated by adenosine, a breakdown product of extracellular ATP and indication of cellular damage, is a G_s_-coupled GPCR that increases cAMP via AC activation, playing a key role in modulating microglial motility and pro-inflammatory responses (Orr et al., 2009). A2AR levels are low in homeostatic microglia but robustly upregulated following inflammatory activation with ibotenate in rats *in vivo* (Colella et al., 2018). A2AR activation induces amoeboid morphology, process repulsion, and reduced surveillance/phagocytosis in activated primary mouse microglia (Orr et al., 2009). A2AR activation also suppresses IL-1β, IL-6 and TNF-α release in rats during excitotoxic and ischemia models *in vivo* (Colella et al., 2018); A2AR antagonism increases these cytokines (Colella et al., 2018). Accordingly, A2AR functions in microglia to limit inflammation and control chemotaxis in retinal and brain neurodegenerative disorders (Santiago et al., 2014).

CBD has recently been demonstrated by two groups to directly and negatively modulate human A2AR expressed in heterologous cells at nanomolar concentrations, potentially as a non-competitive allosteric antagonist (Sánchez-Fernández et al., 2024; Raïch et al., 2023). Crucially, CBD’s inhibition of ATP-induced intracellular calcium increases in cultured N13 microglial cells and in rat primary microglia was blocked by A2AR antagonist ZM 241385, showing microglial evidence that CBD can function through A2AR. However, direct function of CBD through A2AR in microglia or macrophages in inflammation has not been demonstrated.

##### µ-Opioid Receptor (MOR)

MOR is best known as a neuronal GPCR coupled to inhibitory G_ai/o_ proteins and primary receptor for opioid narcotics. In microglia-specific fluorescent MOR reporter mice, ~ 35—50% of microglia across several brain regions were MOR-positive, confirming that MOR is indeed expressed in microglia (Maduna et al., 2019). A recent study showed MOR is upregulated on microglial marker ionized calcium-binding adapter molecule 1 (Iba1)-positive, human induced pluripotent stem cell (iPSC)-derived microglia following activation by LPS treatment or human immunodeficiency virus 1 (HIV-1) infection, and MOR signaling potentiates HIV-induced CXCL10 responses (Skeete et al., 2025). Direct agonism with [D-Ala2, N-MePhe4, Gly-ol]enkephalin (DAMGO) in rat nucleus accumbens increased activated microglia and pro-inflammatory cytokines IL-1α, IL-1β, and IL-6, presumably through microglial MOR (Cuitavi et al., 2023a). Remarkably, microglial MOR is partially responsible for morphine tolerance and withdrawal because microglia-specific MOR knockout delayed tolerance and attenuated withdrawal in mice (Reiss et al., 2022), opening new avenues of study for neuroinflammation and opioid addiction (Cuitavi et al., 2023b).

CBD at micromolar concentrations was a negative allosteric modulator of rat MOR in non-immune cell types and synthetic CBD at micromolar concentrations was also a negative allosteric modulator of human MOR expressed in HEK-293 cells (Kathmann et al., 2006; Bosquez-Berger et al., 2023). However, there are no studies showing that CBD’s anti-inflammatory effects in microglia or macrophages are mediated through MOR, and it remains undemonstrated if other non-psychotropic phytocannabinoids have MOR activity.

##### D2-dopamine receptor (D2DR)

D2DR is a G_i/o_-coupled GPCR with very little known about microglial-specific function or signaling mechanisms. D2DR is not constitutively expressed in healthy resident microglia, but emerges *de novo* on activated microglia and infiltrating macrophages specifically under pathological conditions like cerebral ischemia in mice *in vivo* (Huck et al., 2015). D2/3 agonists enhance nitrite/NO release from LPS/IFNγ-stimulated primary mouse microglia cultures, suggesting D2DR promotes pro-inflammatory effector functions (Huck et al., 2015).

CBD was a partial agonist of rat D2DR in striatal membranes, notably with an IC_50_ of ~ 10—20 nM at D₂High sites obtained from displacement of [3H]domperidone binding (Seeman 2016; Bédard et al., 2007). CBD and CBDV were further identified as a partial agonists of the D2-like dopamine receptor (DOP-3) in *Caenorhabditis elegans* (Shrader et al., 2020). However, there are no studies showing that CBD or CBDV function through D2DR in microglia or macrophages, or inflammation.

##### α₂-adrenoceptor (α₂-AR)

α₂-AR is the receptor for norepinephrine (NE) primarily linked to G_i/o_ signaling, inhibiting AC to reduce cAMP levels (Liu et al., 2016). Upon activation (e.g., by LPS or damage), microglial α₂-AR is upregulated to detect elevated NE to trigger process retraction, reducing ATP/purinergic-directed migration and hyper-ramification toward injury sites, effectively dampening overzealous responses (Gyoneva and Traynelis 2013). Activation of microglial α₂‑AR is anti‑inflammatory; for example, α₂‑AR agonists dexmedetomidine and clonidine significantly reduced the pro‑inflammatory responses in four distinct models: (1) rat primary microglia exposed to LPS *in vitro*, where dexmedetomidine suppressed LPS‑induced TNF‑α, IL‑1β, and IL‑6 secretion and NO/ROS production (Peng et al., 2013); (2) mouse BV‑2 microglia stimulated with LPS, in which dexmedetomidine similarly attenuated LPS‑triggered TNF‑α, IL‑1β, and IL‑6 mRNA and protein along with NLRP3 inflammasome activation, NO, and ROS (Yeh et al., 2018); (3) rat spinal cord microglia *in vivo* following traumatic spinal cord injury, where dexmedetomidine reduced lesion‑site TNF‑α, IL‑1β, and IL‑6 expression and microglial activation, improving functional outcome (Gao et al., 2019); and (4) rat primary microglia subjected to hypoxia/reoxygenation, where both dexmedetomidine and clonidine attenuated ROS formation and TNF‑α expression (Longhitano et al., 2022).

CBG is an exceptionally potent α_2_-AR agonist (EC_50_ = 0.2 nM) in mouse brain membrane cultures (Cascio et al., 2010). While CBG has been shown to modulate neuronal firing through α₂-AR activation in rat neurons (Mendiguren et al., 2023), no data currently exists showing CBG functions through α₂-AR in microglia or macrophages.

##### Other GPCRs

Recent studies showed that CBD was an inverse agonist of human orphan GPCRs (receptors with no confirmed endogenous ligand) GPR3 and GPR12 expressed in heterologous cells (Laun and Song 2017; Laun et al., 2019). Although expressed in microglia, *in vitro* experiments with mouse GPR3 knockout microglia showed that CBD-inhibition of LPS-induced IL‑6 and TNF‑α was independent of GPR3 (Wu et al., 2021). Immunohistochemistry studies of microglia freshly purified from mouse brain showed GPR12 co-localized with Iba1, and GPR12 was upregulated by treatment with cuprizone, a demyelinating toxin (Bédard et al., 2007). However, there is no functional data for GPR12 in microglia or macrophages, and it is unknown whether CBD functions through GPR12 in these cells.

#### TRP channels in microglial-mediated inflammation

TRP channels are a large family of 28 human ion channels corresponding to canonical (TRPC), vanilloid (TRPV), melastatin (TRPM), ankyrin (TRPA), polycystin (TRPP), and mucoliptin (TRPML) classes (Venkatachalam and Montell 2007). TRP channels act as versatile cellular sensors and are major molecular targets of many phytocannabinoids; for example CBD is known to pharmacologically modulate at least 7 TRP channels (TRPV1—4, TRPA1, TRPM8, and TRPC4) (Muller et al., 2018; Etemad et al., 2022; Han et al., 2024). Although the exact number is debated, 10—12 TRP channels are expressed in microglia or upregulated during pro-inflammatory microglial activation, and many function as detectors for disease or injury-related stimuli (Echeverry et al., 2016). TRP channels expressed in microglia with known pharmacological interaction with non-psychotropic phytocannabinoids are reviewed.

##### TRPV1

TRPV1 is a nonselective cation channel most well known as the capsaicin receptor, is robustly expressed in microglia, is a detector for harmful and noxious stimuli including heat, and TRPV1 induction controls microglia activation (Marrone et al., 2017). TRPV1 is directly activated by AEA and other lipid signaling molecules; as such, it is considered a major component of the extended ECS (Fenwick et al., 2017). TRPV1 opening produces rapid intracellular calcium rise, MAPK phosphorylation, and NF-κB activation that enhances IL-6/TNF-α transcription in primary mouse retinal microglia under elevated hydrostatic pressure and primary human microglia via p38/MAPK (Sappington and Calkins 2008; Bhatia et al., 2017). TRPV1 activation notably did not alter expression of pro-IL-1β in primary mouse microglia; TRPV1-mediated calcium influx also activated protein phosphatase 2 A (PP2A) leading to NLRP3 inflammasome activation in primary microglia, and TRPV1 knockout or pharmacological antagonism suppressed caspase1-cleavage and IL-1β/IL-18 maturation (Zhang et al., 2021).

CBD, CBG, CBDV, CBGV and THCV are full but low potency (EC_50_ ~ 1—4 µM) human TRPV1 agonists and prolonged TRPV1 activation by these cannabinoids in heterologous cells desensitizes channels (Iannotti et al., 2014; De Petrocellis et al., 2011; Bisogno et al., 2001). Further, a THCV-botanical drug substance (BDS, e.g. an extract from a cannabis plant chemotype dominant for certain cannabinoid, but containing other cannabis molecules) was the most potent agonist (EC_50_ ~ 200 nM) of human TRPV1 when tested against purified cannabinoids including THCV, suggesting additional regulatory mechanisms like allostery from other molecules in the BDS (De Petrocellis et al., 2011). Activity-dependent desensitization of TRPV1 by these cannabinoids is expected to similarly occur in microglia and other macrophages, leading to a reduction in pro-inflammatory signaling, although it has not been directly shown. Instead, CBD activation of TRPV1 was shown to induce a sustained calcium current and stimulates phagocytosis in BV-2 microglia (Hassan et al., 2014). CBG, CBDV, CBGV and THCV have not yet been shown to function specifically through TRPV1 in microglia.

##### TRPV2

TRPV2 is a non-selective cation channel that is highly expressed in microglia and macrophages and functions as a sensor of cellular stress, membrane stretch, and tissue damage rather than noxious heat (Link et al., 2010; Shibasaki et al., 2010). Unlike TRPV1, TRPV2 does not appear to be gated by endocannabinoids. TRPV2 expression and plasma membrane localization are rapidly and transiently enhanced by inflammatory stimuli such as LPS in primary mouse macrophages (Lévêque et al., 2018), NO in primary mouse microglia (Maksoud et al., 2019) and notably CBD in BV-2 microglia (Hassan et al., 2014). In a mouse vestibular migraine model, TRPV2 expression increased in spinal trigeminal nucleus caudalis microglia, and TRPV2 inhibition in LPS/IFNγ-treated BV-2 microglia shifted polarization from pro-inflammatory to anti-inflammatory, while reducing NLRP3 inflammasome activation and IL-1β, suggesting that TRPV2 functions similarly to TRPV1 in anti-inflammatory signaling (Zhai et al., 2025). TRPV2 also acts as a key regulator of phagocytosis, with TRPV2-mediated calcium influx activating the PI3K pathway and cytoskeletal rearrangement in mouse primary macrophages and microglia (Link et al., 2010; Maksoud et al., 2019).

CBD, CBG, CBDV, CBGV, and THCV were full but low potency rat TRPV2 agonists and desensitizers when expressed in heterologous cells (De Petrocellis et al., 2011; Qin et al., 2008). Further, CBC was revealed as a low potency desensitizer of rat TRPV2, despite having no detectable agonist activity up to several micromolar (De Petrocellis, et al., 2011). Notably, CBD enhanced β-Amyloid (Aβ) phagocytosis via TRPV2 activation in mouse primary and BV-2 microglia (Yang et al., 2022). In contrast, in a rat organotypic hippocampal slice model of stroke, CBD reduced phagocytic Iba + microglia, which was partially TRPV2-dependent (Lana et al., 2022). Therefore, whether TRPV2-dependent CBD signaling is pro- or anti-phagocytic appears model and species dependent. CBG, CBDV, CBGV, and THCV have not been demonstrated to function specifically through TRPV2 in microglia or macrophages.

##### TRPV4

TRPV4 is a nonselective cation channel expressed in microglia that functions primarily as a thermosensor, mechanosensor and osmosensor, responding to changes in membrane tension, shear stress, and extracellular hypotonicity (Nilius et al., 2004). TRPV4 is activated by heat (37—40C) and the epoxyeicosatrienoic acid (EET) metabolites of AA (Watanabe et al., 2003; Nishimoto et al., 2021). Although there is some mixed evidence whether TRPV4 in microglia is pro- or anti-inflammatory, most evidence suggests that TRPV4 signaling is pro-inflammatory. In rat primary microglia induced with LPS, TRPV4 activation by agonist 4α-phorbol-12,13-didecanoate (4α‑PDD) reduced TNF‑α (Konno et al., 2012). In contrast, antagonist RN-1734 showed TRPV4-dependent calcium influx mediated TNF-α and IL-1β expression in LPS-treated primary mouse microglia *in vitro* via NF-κB and similarly reduced pro-inflammatory cytokine release and microglial activation in cuprizone-induced demyelination in mice *in vivo* (Liu et al., 2018). In mice *in vivo* using pilocarpine-induced status epilepticus, TRPV4 antagonist HC-067047 attenuated and agonist GSK1016790A enhanced NF‑κB signaling and TNF‑α, IL‑1β, and IL‑6, although microglial-specific assays were not performed (An et al., 2023). In a similar pilocarpine study, GSK1016790A increased and HC-067047 decreased Iba1 + cell count and expression, NLRP3 inflammasome components NLRP3, apoptosis-related spotted protein (ASC) and caspase-1, and TNF‑α, IL‑1β, and IL‑6 following injection into mouse brains (Wang et al., 2019). Uniquely, TRPV4 also mediates thermosensitive microglial motility in primary mouse microglia and Iba1 fluorescent marker microglia mice *in vivo* (Nishimoto et al., 2021). Activation of TRPV4 triggers calcium influx that promotes microglial process motility and migration through cytoskeletal remodeling via Rho GTPases and actin polymerization (Nishimoto et al., 2021; Redmon et al., 2021).

In rat TRPV4-expressing HEK-293 cells, CBDV and THCV were moderate potency agonists (EC_50_ ~ 0.9–6.4μM) whereas CBG, CBGA, CBGV were more effective at desensitizing TRPV4 channels activated by 4α-PDD, even though they were also low efficacy agonists (De Petrocellis et al., 2012). There is currently no direct evidence, however, that non-psychotropic phytocannabinoids function through TRPV4 in microglia or macrophages.

##### TRPA1

TRPA1 is a nonselective cation channel best known as the receptor for irritant electrophiles such as allyl isothiocyanate (AITC) and cinnamaldehyde (Bautista et al., 2006), with low but detectable expression in microglia where it functions as a sensor of tissue damage-associated molecules and ROS (Yao et al., 2023). TRPA1 is activated by endogenous inflammatory mediators including lipid peroxidation, reactive aldehydes, and oxidants, positioning it as a redox-sensitive effector channel rather than a classical thermosensor (Trevisani et al., 2007; Bautista et al., 2013; Andersson et al., 2008). TRPA1 signaling can be pro- or anti-inflammatory depending on macrophage type and/or activation-dependent (Naert et al., 2021). For example, TRPA1 activation (by AITC or cinnamaldehyde) in LPS‑induced RAW 264.7, J774A.1, and THP‑1 macrophages/monocytes was shown to downregulate MAPK signaling and NO production and TNF‑α and IL‑1β (Radhakrishnan et al., 2023; Chao et al., 2008); whereas, TRPA1 blockade with HC-030031 in hypothermia-induced primary human monocytes decreased TNF-α secretion but increased IL-10 (Billeter et al., 2015). The TRPA1 agonist acreolin increased secretion of CXCL8 (IL-8) in U-937 cells, human alveolar macrophages and THP-1 monocytes via NF-κB activation (Facchinetti et al., 2007). TRPA1 signaling enhanced pro-inflammatory NO, ROS, and IL-17 in BV-2 microglia activated by corticosterone (Lana et al., 2022; Brum et al., 2025). Otherwise, TRPA1 signaling specifically in microglia is unknown and will require further study to elucidate pro- and anti-inflammatory mechanisms. Additionally, reduced inflammation from global TRPA1 knockout is generally ascribed to loss of astrocytic TRPA1 and astrocyte-microglia crosstalk (Xia et al., 2023; De Logu et al., 2017).

CBC and CBD are potent agonists and desensitizers (~ 60—110 nM), and CBG, THCA, CBDA, CBGA, CBDV and THCV are low potency agonists and desensitizers of rat TRPA1 expressed in HEK-293 cells (De Petrocellis et al., 2011; De Petrocellis et al., 2008). Notably, a THCV-BDS was the most potent agonist (EC_50_ ~ 70 nM) of rat TRPA1 in heterologous cells when tested directly against purified cannabinoids including CBC, CBD and THCV, and despite purified THCV being a low potency agonist, suggesting additional TRPA1 regulation by other molecules in the BDS (De Petrocellis et al., 2011). In mouse peritoneal macrophages, CBC reduced LPS‑induced NO production and anti‑inflammatory effects were mimicked by general TRPA1 agonists, but did not show that CBC’s effects were mediated through TRPA1 (Romano et al., 2013). Otherwise, the interplay between phytocannabinoids and TRPA1 signaling in microglia or macrophages is unknown.

##### TRPM7

TRPM7 is a ubiquitously expressed, constitutively active divalent cation channel with a unique C-terminal α-kinase domain (Nadolni and Zierler 2018). It conducts magnesium, calcium, and zinc and is tightly inhibited by intracellular magnesium and Mg-ATP, linking cellular metabolism to ion homeostasis. The α-kinase domain can phosphorylate downstream targets or be cleaved to function as a nuclear signaling molecule. TRPM7 is robustly expressed in primary rat microglia, with large constitutive inward currents and characteristic outward rectification which is inhibited by selective antagonists (Siddiqui et al., 2014). In monocyte-macrophage lineages, TRPM7 expression is upregulated during early inflammation and supports magnesium influx, which in turn drives cleavage of its kinase domain and nuclear translocation (Qiao et al., 2021). Myeloid-wide TRPM7 genetic deletion or inhibitors reduce LPS-induced IL-1β/IL-6 in mouse macrophages and BV2 microglia, suggesting TRPM7 signaling is pro-inflammatory (Busey 2023; Schappe et al., 2018). TRPM7 mediates calcium influx necessary for LPS/TLR4 macrophage activation and canonical nuclear translocation of NF-κB for pro-inflammatory cytokine transcription (Schappe et al., 2018). Further, in a hypomagnesemia epilepsy model, knock-in mice with kinase-dead TRPM7 (K1646R) show normalization of brain NLRP3, ASC, and IL-1β levels and reduced oxidative stress, suggesting kinase activity is also essential for pro-inflammatory signaling (Liu et al., 2023). In mouse bone marrow derived macrophages, TRPM7-dependent magnesium influx caused nuclear accumulation of TRPM7-cleaved kinase fragments localized to histone H3 foci, and enhanced transcription at IL-8 and IκBα promoters via NF-κB activation (Qiao et al., 2021). Together, TRPM7 signaling mediates NF-κB-dependent pro-inflammatory cytokine production through both calcium- and magnesium-dependent mechanisms.

CBGA, CBDA, THCA, THCVA, CBGVA, CBCA, CBG, CBGV, and CBDV were recently found to be inhibitors of human TRPM7 expressed in HEK-293 cells, where CBGA and CBDV were the most potent inhibitors, with IC_50_ values of 1.8 µM and 3.4 µM, respectively (Suzuki et al., 2024). The same study demonstrates that CBGA inhibits native TRPM7 currents in a human B-lymphocyte line (U266).​ In a cisplatin nephropathy model, CBGA administration (10 mg/kg, i.p.) reduced TRPM7 expression in tubular epithelial cells, ameliorated kidney injury, and suppressed mRNA levels of TNF‑α, IL-6, CXCL10, and CCL2, linking CBGA-TRPM7 inhibition to a clear *in vivo* inflammatory context (Suzuki et al., 2023). CBGA or CBDV inhibition of TRPM7 has not yet been examined in microglia or macrophages.

##### TRPM8

TRPM8 is a nonselective cation channel best known as the body’s primary cold sensor, and receptor for “cooling” compounds such as menthol and icilin (McKemy et al., 2002; Chuang et al., 2004). TRPM8 is endogenously expressed and functionally active in primary mouse and BV-2 microglia, where its surface expression is upregulated by LPS signaling and TRPM8 activation itself (Shikha et al., 2023). TRPM8 senses membrane lipids like phosphatidylinositol 4,5-bisphosphate (PIP_2_) to coupling cooling and agonist lipid signals to channel gating and calcium-dependent signaling (Yin et al., 2022; Yin et al., 2019). Because it is directly modulated by endogenous lipids, including AEA, it is also considered a key component of the extended ECS (Storozhuk and Zholos 2018). Crucially, AEA and most phytocannabinoid ligands function as TRPM8 antagonists (De Petrocellis et al., 2008; De Petrocellis et al., 2007). TRPM8 activation and signaling are generally anti-inflammatory in microglia and macrophages. For example, menthol activation of TRPM8 in mouse primary peritoneal and bone marrow-derived macrophages reduced TNF-α and increased IL-10 expression, whereas TRPM8 knockout showed the opposite and implied signaling through p38 and ERK MAPKs (Khalil et al., 2016). TRPM8-dependent calcium signaling in microglia and macrophages appears to engage different signaling microdomains and pathways than TRPV and TRPA1 channels, leading to anti-inflammatory effects (Wu et al., 2023). In a mouse model of PD *in vivo* and LPS challenged BV-2 microglia *in vitro*, TRPM8 activation by icilin reduced microglial activation and release of TNF-α, IL-1β and IL-6 and iNOS expression, with signaling implicated through nuclear factor erythroid 2-related factor 2 (Nrf2)/heme oxygenase 1 (HO-1) upregulation and NF-κB inhibition (Gao et al., 2025). TRPM8 modulation bidirectionally alters cytosolic/lysosomal pH correlations and mitochondrial ROS in BV-2 microglia, with LPS shifting these dynamics, suggesting plasma membrane TRPM8 generates localized calcium near lysosomes/mitochondria for homeostatic tuning (Shikha et al., 2023). Further, TRPM8 activation enhances transferrin-Fe3⁺ uptake and mitochondrial Fe^2^⁺ while preventing overload-induced death in LPS-treated BV-2 microglia, linking calcium influx to anti-inflammatory polarization via lipid peroxidation restraint (Sing et al., 2025). Notably, both TRPM8 activation by menthol and icilin and inhibition by AMTB enhanced early phagocytosis and cytosolic ROS production in primary mouse and BV-2 microglia (Shikha et al., 2023).

CBD, CBG, and THCA are potent antagonists (IC_50_ ~ 70—160 nM) and CBDA, CBGA, THCV, THCVA, CBGV, and CBDV are low-potency antagonists of rat TRPM8 expressed in heterologous cells, and when activated by menthol or icilin (De Petrocellis et al., 2011; De Petrocellis et al., 2008). Notably, a CBG-BDS (IC_50_ ~ 34 nM), THCA-BDS (IC_50_ ~ 56 nM), and THCV-BDS (IC_50_ ~ 20 nM) were more potent antagonists than the purified cannabinoid; whereas, a CBD-BDS (IC_50_ ~ 1.29 µM) was a much less potent antagonist than purified CBD in icilin-activated rat TRPM8, again suggesting complex regulation from additional cannabis constituents in BDS (De Petrocellis et al., 2011). There is currently no data that shows these cannabinoids function through TRPM8 in microglia or macrophages, or that these cannabinoids modulate TRPM8 to impact inflammation, however. Crucially, because these phytocannabinoids inhibit TRPM8 signaling, which is generally anti-inflammatory in microglia, they are expected to be pro-inflammatory through TRPM8.

##### TRPC4

TRPC4 is a non-selective calcium-permeable cation channel expressed broadly in the CNS and periphery, particularly in neurons and astrocytes (Mizoguchi and Monji 2017). TRPC4 mRNA was detected at low-to-moderate levels in cultured rat microglia and in primary human microglia in frontal cortex, temporal cortex, thalamus, and subventricular zone (Lopes et al., 2022; Ohana et al., 2009). TRPC4 is activated primarily downstream of GPCR signaling pathways involving G_q/11_ and G_i/o_ proteins coupled to PLC, which hydrolyzes PIP_2_ to facilitate channel opening (Freichel 2014). It commonly forms functional heterotetramers with TRPC1, TRPC4, and TRPC5, which alter its biophysical properties like calcium permeability and PIP_2_ sensitivity, making isolation of TRPC4-specific effects difficult (Plant and Schaefer 2005). In cultured RAW264.7 macrophages, dual TRPC4/5 inhibition with ML204 increased TNF-α, IL-1β and IL-6 release following LPS stimulation via Akt signaling and NF-κB nuclear translocation (Tao et al., 2020). Similarly, another study showed that ML204 reduced arthritis-associated TNF-α, IL-1β, IL-6, CXCL1 and CCL2 expression in mice *in vivo* (Alawi et al., 2017). However, both studies showed similar effects with TRPC5 knockout and did not test TRPC4-specific involvement. One recent study showed that microglia-specific IL-10 knockout in mice enhanced IL-6, IL-1β, IL-18 and NLRP3 inflammasome components NLRP3, ASC and caspase-1, and reduced global hippocampal TRPC4, although it did not show microglia-specific data (Huo et al., 2021). Recent studies using knockout of all seven TRPC1—7 (HeptaKO) in mice showed increased inflammatory mediator IL-6, CCL4, CCL5, CXCL9 and iNOS expression in adipose macrophages *in vivo* and increased IL-6, IL-12p70, and TNF-α released from HeptaKO bone marrow-derived and peritoneal macrophages in induced colitis, although TRPC4-specific effects were not tested (Lin et al., 2024; Lin et al., 2023). There are currently no primary research studies demonstrating functional roles specific to TRPC4 in microglial or macrophages.

CBD was recently shown to be a moderate-potency inhibitor (IC_50_ = 1.52 μM) of human TRPC4 expressed in HEK-293 cells (Han et al., 2024). It is currently unknown whether other non-psychotropic cannabinoids also inhibit TRPC4. Furthermore, there is currently no evidence that CBD functions through TRPC4 in inflammatory contexts, or specifically in microglia, or macrophages.

#### PPARs in microglial-mediated inflammation

PPARs are nuclear receptors that act as ligand-activated transcription factors that inhibit NF-κB function by direct antagonism and co-factor binding competition. Microglia express three PPARs with different functional specializations: PPARγ, PPARα, and PPARδ/β (Titus et al., 2024). PPARγ is robustly expressed in microglia and acts as the key PPAR transcriptional brake on pro-inflammatory activation (Bernardo and Minghetti 2006). PPARγ agonists suppress pro-inflammatory microglia signaling by downregulating iNOS, TNF-α, IL-1β, and IL-6, while also upregulating CD206, Arg-1, IL-10, and TGF-β, often via direct NF-κB antagonism (Ji et al., 2018). In models of mouse neuropathic pain, rat stroke, and rabbit intraventricular hemorrhage (IVH), PPARγ overexpression or pharmacological activation enhances phagocytosis, and promotes reprogramming of microglia away from pro-inflammatory and toward anti-inflammatory states (Bernardo and Minghetti 2006; Krishna et al., 2021; Li et al., 2021). Notably, AEA is a PPARγ agonist, placing it too as a key component of the extended ECS (Bouaboula et al., 2005). Endogenous signaling lipids such as palmitoylethanolamide (PEA) and oleoylethanolamide (OEA) are established PPARα agonists, including in primary rat microglia (Guida et al., 2017). PPARα activation dampens microglial and macrophage production of TNFα, IL-1β, and IL-6, modulates NO output, and promotes genes involved in fatty acid oxidation and inflammation resolution (Guida et al., 2017; Zhou et al., 2022; Saito et al., 2025). PEA enhances microglial migration and upregulates CB2 expression in cultured rat microglia via PPARα (Guida et al., 2017). PPARδ/β acts as a tonic anti-inflammatory and pro-metabolic regulator, although it is less studied than α/γ (O'Sullivan 2016). PPARδ agonist L-165041 in irradiated BV-2 microglia and PPARδ knockout in primary mouse microglia show PPARδ activity suppresses CCL3, CCL4, CXCL2, TNF-α, IL-1β, IL-6, and iNOS expression, and enhances lipid handling and mitochondrial function and promotes a less neurotoxic state (Doroshenko et al., 2021; Schnegg et al., 2012).

CBD, CBG, and CBC were demonstrated as weak agonists of PPARγ (EC_50_s ~ 10—30 µM) and CBG, CBGA, and CBDA were identified as moderate potency PPARα agonists (EC_50_s ~ 2—13 µM) in transactivation assays in COS-7 cells expressing human ligand-binding domains; activity of CBGA, CBDA, and CBD at PPARγ was confirmed in mouse 3T3-L1 cells whereas CBG, CBGA and CBDA activity at PPARα were confirmed in and human HepG2 cells (D'Aniello et al., 2019; Granja et al., 2012). THCA was identified as a potent PPARγ agonist with receptor displacement studies of rosiglitazone showing an IC_50_ of 0.47 µM, and human ligand binding domain transactivation assays in HEK-293T cells confirmed agonist activity of THCA, CBD, CBG, CBDA, and CBGA where THCA was the most potent (Nadal et al., 2017). Similar transactivation assays showed CBDA was a moderate potency PPARδ/β agonist (EC50 ~ 1.5 µM), where activity in human MDA‑MB‑231 cells blocked by PPARδ/β antagonist GSK3787 or inverse agonist GSK0660 (Hirao-Suzuki et al., 2022). In BV-2 microglia, CBD’s attenuation of LPS-induced IL‑1β, iNOS and NLRP3 expression and caspase-1 activity was partially mediated by PPARγ activation, as it was partially blocked by antagonist GW9662 (da Silva et al., 2024). In mice *in vivo*, CBD (10 mg/kg, i.p.) reduced Aβ-induced neuroinflammatory IL‑1β, TNF‑α and NO in hippocampus, which was blunted by GW9662, also suggesting CBD’s anti-inflammatory effects were partially PPARγ-dependent (Esposito et al., 2011). THCA (10 mg/kg/day, p.o.) similarly reduced TNF-α and IL-6 expression in mouse brains treated with mitochondrial toxin 3-nitropropionic acid (3-NPA), and was partially PPARγ-dependent (Nadal et al., 2017). *In vivo* studies have not demonstrated that THCA-PPARγ modulation of cytokine production is specific to microglia, however. Furthermore, CBG, CBC, CBDA, and CBGA have not been shown to directly function through PPARs in microglia or inflammation.

#### Anti-inflammatory evidence of non-psychotropic phytocannabinoids in microglia and macrophages without clear MOA

There is substantial additional evidence that several non-psychotropic phytocannabinoids reduce microglia and macrophage activation and pro-inflammatory responses without a determined or explicitly tested MOA. Several recent examples are highlighted here.

##### CBD

CBD has the most additional evidence of microglial anti-inflammatory activity. In human microglial cell line HC69.5, CBD reduced HIV-infection-induced pro-inflammatory cytokines and chemokines IL‑6, IL‑8, IL‑1β, MCP‑1, GM‑CSF, CXCL1 and CXCL10, and NLRP3 gene expression and caspase-1 activity that was independent of CB receptors (Yndart Arias et al., 2023). Synthetic CBD’s MOA was untested in a cultured primary mouse microglial study that showed CBD reduced LPS-induced glucose-driven NADPH regeneration, oxidative stress, and TNF‑α and IL‑1β release that was NF-κB-dependent (dos Santos‐Pereira et al., 2020). A study using GPR3 knockout mice also showed synthetic (-)-CBD inhibition of LPS-induced IL-1β and TNF-α in primary wild type and knockout microglia, but did not test MOA (Wu et al., 2021). A CBD-BDS with terpenes reduced LPS‐induced upregulation of the pro‐inflammatory cytokines IL‐1β, IL‐6, and TNF‐α in BV-2 microglia, and was only partially dependent on CB2 revealed by antagonist SR144528 (Borgonetti et al., 2022). Similarly, CBD blocked inflammation in LPS-stimulated inflammation in RAW 264.7 macrophages and a λ-carrageenan-induced mouse model (10—20 mg/kg, i.p.) via NF-κB inhibition (Kim et al., 2023b).

##### CBG

Several primary studies on CBG have documented its anti-inflammatory effects in macrophages and microglia without confirming or testing MOAs. CBG reduced LPS-induced iNOS and TNF-α expression in BV-2 microglia and primary rat mixed glial cultures with no MOA tested (Fleisher-Berkovich et al., 2023). Surprisingly, a recent study also in BV-2 microglia showed that 10 nM hemp-derived CBG alone upregulated caspase-1 expression higher than a combination of LPS/Nigericin, suggesting that CBG may have underappreciated pro-neuroinflammatory effects (Dos Santos et al., 2026). Regardless, CBG blocked iNOS and TNF‑α expression in LPS-stimulated RAW 264.7 macrophages; similarly, CBG (10—20 mg/Kg, i.p.) decreased iNOS, TNF-α, IL-1β, and IL-6 expression in a λ-carrageenan-induced mouse model via NF-κB inhibition (Kim et al., 2025). Furthermore, in an *in vitro* model of neuroinflammation, NSC34 motor neuron death was decreased when exposed to medium from LPS-stimulated RAW264.7 macrophages that were pre-treated with CBG (Gugliandolo et al., 2018).

##### CBC and THCV

There are also several recent studies showing unmapped CBC and THCV anti-inflammatory effects in macrophages without explicitly tested MOAs. CBC blocked iNOS, IL-1β, IL-6, and TNF-α expression in LPS-stimulated RAW 264.7 macrophages and a λ-carrageenan-induced mouse model (10—20 mg/kg, i.p.) via NF-κB inhibition (Hong et al., 2023). In a separate study, THCV (0.3—5 mg/kg, i.p.) also reduced inflammatory paw edema and thermal hyperalgesia in a λ-carrageenan-induced mouse model but did not investigate isolated macrophages or MOA (Bolognini et al., 2010). Similarly, both CBC and THCV downregulated IL‑1β and the IL-6/STAT-3 pathway and NF‑κB phosphorylation in LPS-treated THP-1 macrophages (Gojani et al., 2023). Moreover, CBC reduced NO in LPS-stimulated murine peritoneal macrophages (Romano et al., 2013). In a mouse model of gut inflammation, CBC (10—20 mg/kg, i.p.) reduced croton‑oil‑induced hypermotility in the small intestine and colon and was not modified by CB1, CB2, or TRPA1 antagonists (Izzo et al., 2012). Notably, unlike CBD and CBG, there are no studies that show the effects of CBC or THCV directly on microglial inflammation responses.

### Non-psychotropic phytocannabinoids as microglial-mediated neurodegenerative disease therapeutics

Table 1 lists pre-clinical neurodegenerative disease models for AD, PD, MS, and HD treated with non-psychotropic phytocannabinoids and the results of microglial-specific assays; Table 2 lists completed, ongoing, and planned clinical trials on AD, PD, MS, and HD with non-psychotropic phytocannabinoids. Both are discussed in more detail below.

**Table 1:** Preclinical AD, PD, MS, and HD disease models with non-psychotropic phytocannabinoid treatment and microglial assays

Disease model	Disease induction	Phytocannabinoid treatment	Microglial effects (assayed readouts and direction of change vs. disease)	Study
AD	Aβ i.c.v. injection in mice	CBD (25 mg/kg, i.g.)	Hippocampal expression: ↓TNF-α, ↓CCL2; Hippocampus and cortex: ↓Iba1 + microglial density	Chen et al., 2023
AD	Aβ applied to primary mouse microglia *in vitro*	CBD (5 µM)	Primary mouse microglia: ↓IL-1β, ↑IL-6, ↑IL-4, ↑TRPV2, ↑TREM2;↑phagocytosis of fluorescent Aβ	Yang et al., 2022
AD	5xFAD transgenic mice (Aβ overdeposition) and primary microglia from 5xFAD mice	CBD (10 mg/kg, i.p.)	Hippocampus and cortex: total Iba1 + microglial number unchanged, ↑Arg-1 + microglia, ↑iNOS; cultured 5xFAD microglia: ↓IL-1β, ↑IL-10	Raïch et al., 2025
PD	6-OHDA lesion of nigrostriatal pathway in rats	CBD (10 mg/kg, i.p.)	Substantia nigra pars compacta and striatum: CD11b + microglial density and polarization (CD11b/CD32 + and CD11b/CD206 +) unchanged; ↑microglial endpoints	Giuliano et al., 2021
PD	Rotenone-induced Parkinsonism in rats	CBD nanoemulsion (CBDne, 5 mg/kg, p.o.)	Striatum and duodenum: ↓iNOS, nNOS, NO; ↓Iba1 + microglial immunoreactivity	Dos Santos et al., 2025
MS	MOG-EAE in mice	CBD (5 mg/kg, i.p.)	Spinal cord: ↓Iba1 + immunoreactivity	Kozela et al., 2011
MS	MOG-EAE in mice	CBD (5 mg/kg, i.p.)	Spinal cord: ↓Iba1 + immunoreactivity	Li et al., 2022
MS	MOG-EAE in mice	CBD (5 mg/kg, i.p.)	Spinal cord mRNA: ↓TNF‑α, ↓IFN‑γ, ↓IL‑17	Rahimi et al., 2015
MS	Theiler’s virus–induced demyelinating disease in mice	CBD (5 mg/kg, i.p.)	Prefrontal cortex: ↓Iba1, ↓IL-1β, ↓CCL2, ↓CCL5; spinal cord: ↓Iba1, ↓TNF‑α, ↓IL-1β; cerebral cortex: ↓Iba1	Mecha et al., 2013
MS	Cuprizone-induced demyelination in mice	CBD (5 mg/kg, i.p.)	Corpus callosum: ↓Iba1 + microglial density, ↓MDA, ↑GSH, ↑CAT, ↑SOD	Sajjadian et al., 2017
MS	MOG-EAE in mice	CBG (10 mg/kg, i.p.)	Spinal cord: ↑Iba1 + microglial density	Fleisher-Berkovich et al., 2023
HD	3-NP toxin model in mice	CBG (10 mg/kg, i.p.)	Striatum expression: ↓Iba1 + microglial density; ↓TNF-α; ↓IL-4; ↓iNOS, ↑GSH; striatum activity: ↑CAT, ↑SOD	Valdeolivas et al., 2015

**Table 2:** AD, PD, MS, and HD clinical trials with non-psychotropic phytocannabinoids

Disease	Phytocannabinoid treatment (dose, duration, n of treatment arm)	Outcome (assays and direction of change)	Study
AD	CBD (titrated to ~ 600 mg/day; 6 weeks; *n* = 8)	BPSD domains (agitation, anxiety, apathy, irritability, caregiver distress): signal of improvement vs placebo; cognitive outcomes: no significant difference (trial not powered for cognition)	Velayudhan et al., 2024
AD	Low-dose THC:CBD (0.350 mg THC + 0.245 mg CBD daily; 26 weeks; *n* = ~ 15)	MMSE: higher scores in cannabinoid group vs placebo, suggesting stabilization or improvement of global cognition; other symptom domains exploratory. CBD not tested as monotherapy	Cury et al., 2025
AD	CBD (titrated to ~ 45 mg/day, 10 weeks, *n* = ~ 40)	Planned outcomes: agitation in AD (CMAI), caregiver burden, quality of life, safety/tolerability	NCT04436081 (active/unknown)
AD	CBD (200 mg/day, 24 weeks, *n* = ~ 118)	Planned outcomes: neurocognitive function, biomarkers of AD progression, pain, sleep, anxiety and stress, and inflammation and oxidative stress biomarkers	NCT05822362 (active)
AD	CBD (up to 800 mg/day, 19 weeks, *n* = ~ 50)	Planned outcomes: agitation severity (CMAI), behavioral symptoms, cognition (MMSE), nutrition and weight, pain	NCT06014424 (active)
AD	CBD (proprietary, 8 weeks, *n* = 12)	Planned outcomes: anxiety (GAD-7), safety, delirium, and agitation	NCT04075435 (active)
PD	CBD (75 or 300 mg/day, 6 weeks, *n* = 7 per dose)	UPDRS motor: no significant improvement vs placebo; PDQ-39 quality of life: significant improvement vs placebo; plasma BDNF or ^1^H‑MRS measures: unchanged	Chagas et al., 2014
PD	CBD-enriched sublingual product (~ 26 mg CBD/day, 12 weeks, *n* = 27	MoCA: improved naming scores; elevated alkaline phosphatase; no change: motor function (UPDRS), mood, inflammatory blood markers	Mitarnun et al., 2025
PD	Epidiolex®-formulation CBD (titrated to 20—25 mg/kg/day, 13 weeks, *n* = 13)	UPDRS: improvement in some motor measures; elevated cholestatic liver enzymes	Leehey et al., 2020
PD	CBD (titrated to 2.5 mg/kg, 3 weeks, *n* = 30)	UPDRS motor scores: no significant benefit vs placebo; safety acceptable	NCT03582137 (completed)
PD	CBD (100 mg/ml, 300 mg/ml, 400 mg/ml, 12 weeks, *n* = ~ 11 per dose)	Planned outcomes: motor symptoms, quality of life, mood, sleep, cognition, and pain	NCT06629389 (active)
PD	CBD (MTD up to 800 mg/day, 12 weeks, *n* = ~ 50)	Planned outcomes: psychosis severity	ISRCTN87895237 (completed, unpublished)
MS	CBD (up to ~ 45 mg/day; 6 weeks, *n* = 31)	Neuropathic pain: no significant improvement vs placebo; spasticity: no significant improvement; safety: generally well tolerated at tested dose	Hansen et al., 2023
MS	CBD (titrated from ~ 5 to 80 mg/day over ~ 4 weeks; *n* = 24)	Spasticity severity: no significant reduction vs placebo; some measures of walking speed and pain modestly favored CBD, not primary endpoint	Mousavi et al., 2025
MS	CBD (50 mg/day, 15 weeks, *n* = unknown)	Planned outcomes: urinary symptoms, bladder function, quality of life	NCT06261489 (active)
MS	CBD (Epidiolex® 200 mg/day, 12 weeks, *n* = ~ 62)	Planned outcomes: sleep quality and pain scores; secondary: safety	NCT05269628 (active)
MS	CBD (200 mg/day, 16 weeks, *n* = ~ 40)	Planned outcomes: spasticity severity; secondary: pain, sleep, physical function, quality of life and safety	NCT05092191 (active)
HD	CBD (10 mg/kg/day (~ 700 mg/day average), 6 weeks, *n* = 15)	Chorea; motor, cognitive, psychiatric HD symptoms: no significant improvement vs placebo; safety: CBD generally well tolerated at this dose	Consroe et al., 1991

#### AD

AD is the most common neurodegenerative disorder and leading cause of dementia, defined clinically by progressive cognitive decline, memory loss, executive dysfunction, language impairment, and behavioral disturbances such as apathy or agitation (Scheltens et al., 2021). AD is characterized by selective neuronal loss and synaptic dysfunction primarily in hippocampus-dependent memory circuits and neocortical association areas, leading to impaired learning, episodic memory, and higher-order cognition as these networks degenerate (Scheff and Price 2006). AD is multisystem, with early involvement of medial temporal lobe structures (entorhinal cortex, hippocampus) followed by neocortical spread, and non-cognitive features including sleep disruption, mood changes, and visuospatial deficits that can emerge alongside or prior to overt amnesia (Jack et al., 2018). Only ~ 1—5% of AD is familial due to highly penetrant mutations in amyloid precursor protein (APP), presenilin (PSEN) 1, or PSEN2, while > 70 common genetic risk variants (led by apolipoprotein E (APOE)-ε4) and dozens of rare variants explain ~ 20—40% of heritable late-onset risk (Cacace et al., 2016; Kunkle et al., 2019; Bellenguez et al., 2022).

A key histopathological hallmark of AD is extracellular accumulation of Aβ peptides into neuritic plaques, predominantly in hippocampus and neocortex, which is thought to initiate a pathogenic cascade through prion-like seeding and spread across vulnerable circuits (Hardy and Higgins 1992). The second core feature is intraneuronal aggregation of hyperphosphorylated microtubule-associated protein tau into neurofibrillary tangles (NFTs), with tangle burden strongly correlating with cognitive decline and synaptic loss (Arriagada et al., 1992). Aβ derives from sequential β- and γ-secretase cleavage of APP; soluble Aβ oligomers disrupt synaptic plasticity and trigger tau pathology, while fibrillar Aβ recruits microglia and astrocytes to plaques (Selkoe 2008).

Chronic microglia-mediated neuroinflammation is now viewed as a key contributor to the initiation and progression of AD. Multiple lines of evidence support a model in which Aβ aggregates and NFTs act as key triggers of microglial activation in AD. Extracellular Aβ species (oligomers, fibrils, plaques) engage TLR2/4/6 and receptor for advanced glycation end products (RAGE) on microglia, activating NF-κB signaling, priming, and NLRP3 inflammasome assembly with subsequent release of pro-inflammatory cytokines, chemokines, and ROS/NOS that amplify synaptic toxicity and tau spread (Heneka et al., 2015). Tau oligomers/fibrils similarly activate integrin-CD47/TREM2 signaling and spleen tyrosine kinase (SYK)-NLRP3 pathways in microglia, promoting a vicious cycle of cytokine production, impaired phagocytosis of pathological proteins, and synaptic pruning that contributes to a toxic microenvironment for vulnerable cholinergic and glutamatergic neurons (Huang et al., 2023).

Several *in vitro* and *in vivo* pre-clinical AD studies using Aβ challenge have shown or inferred microglial-mediated anti-inflammatory effects of CBD. A single intracerebroventricular (i.c.v.) injection of CBD (2.5 µg/5 µl) prevented mouse Aβ i.c.v. injection-induced IL-6 expression and spatial memory deficits, which indirectly inferred microglial modulation (Martín-Moreno et al., 2011). Similarly, CBD (2.5—10 mg/kg, i.p.) after hippocampal Aβ injection in mice reduced iNOS and IL-1β expression, but did not specifically study microglia (Esposito et al., 2007). Furthermore, intragastric (i.g.) CBD treatment (25 mg/kg) reduced i.c.v. Aβ-induced hippocampal expression of TNF-α and CCL2 and Iba + microglial density in cortex and hippocampus, suggesting direct microglial involvement in CBD’s therapeutic activity (Chen et al., 2023). Crucially, a recent study showed CBD directly attenuated microglia-mediated Aβ inflammation *in vivo*. Using 5xFAD transgenic mice, a genetic AD model with abundant Aβ deposition, CBD (10 mg/kg, i.p.) did not change total Iba1 + microglia, but did increase Arg-1 + microglial cell density and iNOS expression and resulted in significant improvements in both short- and long-term spatial memory; CBD treatment reduced IL‑1β and increased IL-10 cytokine expression from primary microglia cultured from 5xFAD mice (Raïch et al., 2025). CBD (5 µM) increased expression of TRPV2 and phagocytosis-related marker TREM2 and decreased IL‑1β, but paradoxically increased IL-6 and IL-4 expression in Aβ-treated primary mouse microglia and enhanced phagocytosis of fluorescently labeled Aβ via TRPV2/Akt signaling (Yang et al., 2022). We are unaware of tauopathy or tau oligomer/fibril AD models which investigated effects of non-psychotropic phytocannabinoids with microglial assays.

Non-psychotropic phytocannabinoids other than CBD have not been tested or demonstrated microglial-specific effects in preclinical AD models; nonetheless, CBDA, THCA, and CBGA have shown anti-inflammatory effects that could infer microglial modulation. Hippocampal injections of CBDA (6 μM, 3 μL) and THCA (12 μM, 3 μL) were tested alongside hippocampal Aβ-injected mice, where both cannabinoids reduced hippocampal Aβ and p-tau accumulation and improved cognitive performance (Kim et al., 2023a). Further, repeated treatment with CBDA or CBGA (10 mg/kg, i.p.) improved intraventricular Aβ-induced cognitive deficits and restored hippocampal long-term potentiation and TRPM7 expression in an AD mouse model (Vitale et al., 2025).

Two small completed clinical trials of CBD in AD-related dementia have been generally positive. In a phase 2a feasibility, single-site, randomized, double-blind, placebo-controlled, parallel-group trial in 16 patients with AD and behavioral and psychological symptoms of dementia (BPSD) where participants (*n* = 8) received oral CBD (titrated up to 3 × 200 mg/day) or placebo over 6 weeks, there were signals of potential benefit on domains such as agitation, anxiety, apathy, irritability, and caregiver distress compared to placebo but the trial was not powered to detect cognitive efficacy (Velayudhan et al., 2024). In another phase 2 randomized, double-blind, placebo-controlled, parallel-group clinical trial in 29 patients (60—80 yrs) with AD-associated dementia randomized for treatment and placebo, low-dose THC:CBD extract (0.350 mg THC + 0.245 mg CBD daily) versus placebo over 26 weeks, Mini-Mental State Exam (MMSE) scores were significantly higher in the cannabinoid group versus placebo, suggestive of stabilization or improvement in global cognitive performance (Cury et al., 2025). This trial was included in the current review because it dosed THC at a concentration well below the accepted psychotropic dose; however, CBD and THC were not trialed as monotherapies, and CBD’s contribution to clinical outcome is unknown. However, based on a recent mouse study with low dose synthetic cannabinoids, it seems likely that THC is either necessary or sufficient at this low dose for AD therapeutic action; in an AD model using 5xFAD mice, synthetic THC alone or combined CBD:THC (CBD (0.273 mg/kg), THC (0.205 mg/kg, i.p.)), but not CBD alone provided therapeutic outcomes (Chen et al., 2023; Arnanz et al., 2024). NCT04436081 is an estimated completed phase 2, randomized, double‑blind, placebo‑controlled, crossover trial in patients with AD dementia or mixed AD dementia and agitation using CBD (titrated to ~ 45 mg/day) for 10 weeks and targeted enrollment of 40, with primary endpoint Cohen-Mansfield Agitation Inventory (CMAI) and secondary endpoints of caregiver burden, quality of life, and safety/tolerability; however, results have not been published 2.5 + years following completion and it is marked as unknown status. NCT05822362 is an active phase 2, double‑blind, randomized, placebo‑controlled, parallel‑group trial in individuals diagnosed with mild cognitive impairment (MCI) at risk for AD (aged 55—70 +) using 200 mg/day CBD, estimated enrollment of 236, duration of 24 weeks, and planned primary endpoints of neurocognitive function, biomarkers of AD progression (plasma p‑tau181, Aβ42/Aβ40 ratio, neurofilament light) and secondary outcomes of pain, sleep, anxiety and stress, and inflammation and oxidative stress biomarkers. NCT06014424 is an active phase 2, double‑blind, randomized, placebo‑controlled, crossover trial in patients with AD and clinically significant agitation using CBD up to 800 mg/day for 19 weeks, targeted enrollment of 50, and primary endpoints of agitation severity (CMAI) and secondary outcomes of behavioral symptoms, cognition (MMSE), nutrition and weight, and pain. NCT04075435 is a phase 1, open‑label, 8‑week single‑arm trial in older adults (55–90 years) with MCI or AD‑related dementia and clinically significant anxiety or behavioral symptoms, using a proprietary high CBD/low THC formulation and 12 participants with primary outcomes in anxiety measured by the Generalized Anxiety Disorder 7 (GAD-7) scale and secondary endpoints of safety, delirium, and agitation. We are unaware of any non-psychotropic phytocannabinoids other than CBD that have been tested in completed, ongoing, or planned AD clinical trials.

#### PD

PD is the second most common neurodegenerative disorder, defined clinically by bradykinesia, rigidity, rest tremor, and postural instability (Bloem et al., 2021). PD is characterized by progressive degeneration of tyrosine hydroxylase (TH)-positive dopaminergic neurons in the substantia nigra pars compacta, leading to striatal dopamine depletion and the cardinal motor syndrome once enough of these neurons and their terminals are lost. The disease is multisystem, with involvement of additional central and peripheral autonomic and sensory structures that underline nonmotor features such as hyposmia, sleep disturbance, constipation, and cognitive impairment that can precede motor onset by years. Only ~ 10% of PD is caused by single gene variants including α-synuclein, leucine-rich repeat kinase 2 (LRRK2), and parkin, whereas over 90 additional genetic risk variants explain ~ 20—30% of heritable non-monogenic PD (Dehestani et al., 2021; Trevisan et al., 2024; Shi et al., 2024).

A central histopathological hallmark is abnormal aggregation of α-synuclein proteins into Lewy bodies in vulnerable neuronal populations, which is thought to spread in a prion-like fashion through interconnected brain regions (Jan et al., 2021). Chronic microglia-mediated neuroinflammation is now viewed as a key contributor to the initiation and progression of this degeneration rather than a purely epiphenomenal response (Isik et al., 2023). Multiple lines of evidence support a model in which misfolded or extracellular α-synuclein acts as a key trigger of microglial activation in PD. Extracellular α-synuclein species can engage TLRs on microglia and activate NF-κB signaling, leading to priming and activation of the NLRP3 inflammasome and subsequent release of pro-inflammatory cytokines that promote neuronal injury and death (Li et al., 2021b). Chronic production of cytokines, chemokines, ROS/NOS, and complement components by persistently activated microglia contributes to a toxic microenvironment for dopaminergic neurons, which are particularly vulnerable due to their high metabolic demand and intrinsic oxidative stress (Ho 2019).

To date, pre-clinical research models of PD have mostly indirectly inferred microglial-mediated changes due to phytocannabinoid treatments. For example, in a study that used 1-methyl-4-phenyl-1,2,3,6-tetrahydropyridine (MPTP) systemic exposure to induce dopaminergic neuron injury and death in mice, CBD improved functional performance and reduced TNF-α, IL-1β, IL-6 and NLRP3/caspase-1/IL-1β inflammasome components, and increased IL-10, but direct microglial metrics were not assessed (Wang et al., 2022a). We are aware of only two studies that directly assessed microglia in a PD preclinical model treated with non-psychotropic phytocannabinoids. The first study used a rotenone-induced rat model of PD which leads to elevated α-synuclein in brain and peripheral tissues, and showed a nanodelivery formulation of CBD (CBDne, 5 mg/kg, p.o.) reduced rotenone-induced increases in α-synuclein accumulation in the striatum and duodenum, attenuated iNOS, nNOS and NO increases, and normalized Iba1 levels (Dos Santos et al., 2025). In the second study, CBD (10 mg/kg, i.p.) attenuated 6-Hydroxydopamine (6-OHDA) lesion-induced striatal terminal loss, dopaminergic cell loss, increased number of microglial endpoints, but did not alter CD11b + microglial density or pro- or anti-inflammatory polarization (CD11b/CD32 + and CD11b/CD206 +, respectively) (Giuliano et al., 2021). In the same study, CBD significantly reduced reactive astrocytic markers, suggesting neuroprotective roles were independent of microglial modulation. These mixed results suggest that CBD’s action on microglia differs between PD induction models. Besides CBD, THCV is the only other non-psychotropic phytocannabinoid to have been tested in a preclinical PD model. In this study, THCV (2 mg/kg, i.p.) reduced dyskinesia in L-3,4-dihydroxyphenylalanine (L-DOPA)-treated Pitx3^ak^ mutant mice, but did not investigate microglia or inflammation (Espadas et al., 2020).

CBD has been evaluated in several recent PD clinical trials. Unfortunately, acute CBD at liver-safe doses generally does not appear to be effective at treating PD motor and cognitive symptoms. In a randomized, double-blind, placebo-controlled, 3-group trial with 21 PD patients, CBD 300 mg/day for 6 weeks (*n* = 7) did not improve motor symptoms measured by the Unified Parkinson’s Disease Rating Scale (UPDRS) or plasma BDNF or 1H‑MRS measures, but did significantly improve reported quality of life measured by Parkinson’s Disease Questionnaire (PDQ‑39) (Chagas et al., 2014). In a 12-week randomized, double-blind, placebo-controlled, parallel-group trial of 27 treated PD patients, sublingual CBD-enriched product of ~ 26 mg CBD/day showed no significant differences in overall cognition (except improved naming scores) using the Montreal Cognitive Assessment (MoCA), nor did it improve motor function (UPDRS), mood, or inflammatory blood markers compared with placebo; however, CBD elevated plasma alkaline phosphatase levels (Mitarnun et al., 2025). CBD was associated with motor score improvement (UPDRS), however, in an open‑label, dose‑escalation pilot study in adults with PD and substantial rest tremor and 13 completers treated with high-dose, CBD brand Epidiolex® titrated to 20—25 mg/kg/day (Leehey et al., 2020). However, in this same study 5/13 of high-dose (20—25 mg/kg/day) patients had elevated cholestatic liver enzymes, which all resolved post-discontinuation. In a follow-up phase 2a, randomized, double‑blind, placebo‑controlled, crossover trial in PD patients with rest and intention tremor (NCT03582137) included testing purified CBD (not Epidiolex®) for PD tremor and psychosis with titration up to 2.5 mg/kg/day (much lower than 20—25 mg/kg/day) in 30 participants did not show benefit versus placebo. NCT06629389 is an ongoing phase 2 randomized, double-blind, placebo-controlled, parallel-group trial evaluating 100 mg/ml, 300 mg/ml and 400 mg/ml doses of Kanbis® brand CBD in PD symptoms, 88 enrolled participants randomized into 4 groups, 12 week duration and proposed endpoints of motor symptoms, quality of life, mood, sleep, cognition, and pain. ISRCTN87895237 is a completed two-part clinical trial evaluating CBD for PD psychosis that is not yet published. In part 1, an open-label dose escalation was proposed to find the maximum tolerated dose (MTD) in 200 mg/day increments, up to 800 mg/day and 3 participants per cohort. Part 2 is a randomized, double‑blind, placebo‑controlled, parallel‑group trial at the established MTD for 12 weeks with ~ 100 total randomized participants. Like AD, we are unaware of any clinical trial for PD with non-psychotropic phytocannabinoids other than CBD.

#### MS

MS is a chronic, immune-mediated, demyelinating disease of the CNS affecting ~ 2.8 million people worldwide (Walton et al., 2020). Clinically, MS is characterized by a highly variable course with episodes of neurological dysfunction (relapses) and progressive accumulation of disability. Symptoms can include sensory disturbances, limb weakness, spasticity, visual impairment (optic neuritis), bladder/bowel dysfunction, fatigue, cognitive decline, and mood changes (Thompson et al., 2018; Reich et al., 2018). Disease onset most commonly occurs in early adulthood (20—40 years), with a female predominance of ~ 3:1 in relapsing forms (Lassmann 2018). MS is heterogeneous, encompassing several phenotypes: relapsing–remitting MS (RRMS), secondary progressive MS (SPMS), and primary progressive MS (PPMS). While approximately 85% of patients initially present with RRMS, many transition to SPMS over 10—20 years, characterized by gradual accumulation of disability independent of relapses (Thompson et al., 2018; Reich et al., 2018). Genetics contribute to susceptibility, with over 200 loci identified, including variants in major histocompatibility complex, class II, DR beta 1 (HLA-DRB1), but the majority of cases are polygenic and influenced by environmental factors such as vitamin D deficiency, viral infections (notably Epstein-Barr Virus), and smoking (Consortium 2019; Bjornevik et al., 2022; Olsson et al., 2017).

A central hallmark of MS is focal demyelination and neurodegeneration, accompanied by chronic microglial activation and neuroinflammation (Guerrero and Sicotte 2020; Kutzelnigg et al., 2005). In the relapsing phase, disease is dominated by adaptive immune cell infiltration, with autoreactive T and B cells crossing the blood–brain barrier and initiating demyelinating lesions (Hemmer et al., 2015). However, in progressive MS, chronic CNS-resident inflammation, largely driven by activated microglia and CNS-intrinsic macrophages, predominates and drives axonal loss and neurodegeneration even in the absence of peripheral immune cell influx (Reynolds et al., 2011).

Microglia in MS demonstrate diverse phenotypes: they can be neurotoxic, producing pro-inflammatory cytokines, ROS/NOS, and complement components that damage myelin and axons, or neuroprotective, promoting remyelination and debris clearance (Guerrero and Sicotte 2020; Miron et al., 2011). Chronic activation of microglia has been observed in normal-appearing white matter (NAWM) and cortical gray matter in both human post-mortem studies and *in vivo* imaging using translocator protein positron emission tomography (TSPO-PET) tracers (Zrzavy et al., 2017; Rissanen et al., 2018; van der Poel et al., 2019). Persistent microglial inflammation is now considered a key contributor to disease progression, particularly in SPMS and PPMS. Extracellular factors such as myelin debris, oxidized lipids, and damage-associated molecular patterns (DAMPs) can activate microglia via TLRs, inflammasome pathways, and NF-κB signaling, amplifying neuroinflammation in MS (Yong 2022). The resulting milieu of cytokines, chemokines, ROS, and complement promotes oligodendrocyte injury, neuronal and axonal degeneration, and ultimately contributes to irreversible clinical disability.

CBD has been tested in preclinical MS models, primarily experimental autoimmune encephalomyelitis (EAE), with direct evidence of anti-inflammatory microglial effects. CBD (5 mg/kg, i.p.) administration during EAE induction in mice by myelin oligodendrocyte glycoprotein (MOG) reduced microglial-specific Iba1 + immunoreactivity and CD3 + infiltrating T-cell density, and decreased disease severity and axonal damage (Kozela et al., 2011). In another MOG-EAE mouse study, CBD (5 mg/kg, i.p.) similarly reduced Iba1 expression and reduced disease severity, which was blocked by Mitofusin 2 knockdown (Li et al., 2022). In a third MOG-EAE mouse study, CBD (5 mg/kg, i.p.) decreased pro-inflammatory cytokine TNF‑α, IFN‑γ, and IL‑17 mRNA expression and reduced the severity of neurobehavioral scores, although cytokine expression did not delineate source between microglia and infiltrating macrophages (Rahimi et al., 2015). Interestingly, early oral CBD (75 mg/kg) treatment prevented MOG-EAE-induced Splenic IFN‑γ + T cells but not IL-17A + T cells or IL-17A production after *ex vivo* restimulation, suggesting the CBD impact on IL-17 mentioned above may indeed be microglial-mediated (Nichols et al., 2021). In a Theiler’s murine encephalomyelitis virus-induced mouse EAE model, CBD (5 mg/kg, i.p.) decreased disease severity and reduced IL-1β, CCL2, and CCL5 in the prefrontal cortex and lowered TNF‑α and IL 1β in the spinal cord; CBD also reduced Iba1 expression in the prefrontal cortex, spinal cord, and cerebral cortex, the latter of which was partially A2AR-mediated because it was incompletely blocked by ZM 241385 (Mecha et al., 2013). Using a cuprizone-induced demyelination MS mouse model, CBD (5 mg/kg, i.p.) reduced demyelination, oxidative stress measured by malondialdehyde (MDA), glutathione (GSH), catalase (CAT) and superoxide dismutase (SOD), and Iba1 + microglia accumulation in the corpus callosum (Sajjadian et al., 2017). Four injections of CBG (10 mg/kg, i.p.) also decreased behavioral severity and neuron death in MOG-EAE mice, but in contrast to CBD studies, surprisingly increased Iba1 + microglial cell count in spinal cord sections (Fleisher-Berkovich et al., 2023). These results suggest that CBD and CBG may have differing effects on MS. We are unaware of any other non-psychotropic phytocannabinoids tested in preclinical MS models.

CBD monotherapy has been evaluated in only two MS clinical trials, both of which failed to show clinical improvement. In a four‑armed, multicenter, randomized, double‑blinded, placebo‑controlled, parallel‑group trial in patients with MS or spinal cord injury (SCI) and central neuropathic pain and/or spasticity, 31 patients assigned to the CBD monotherapy arm (up to ~ 45 mg/day) showed no significant improvement in neuropathic pain or spasticity (Hansen et al., 2023). In single‑center, randomized, double‑blind, placebo‑controlled, parallel‑group trial in MS patients with spasticity‑related gait problems escalating CBD doses from ~ 5 mg/day up to ~ 80 mg/day over ~ 4 weeks in 24 patients did not reduce spasticity severity, though some measures of walking speed and pain favored CBD (Mousavi et al., 2025). NCT06261489 is an active pilot, phase II, randomized, double‑blind, two‑arm, placebo‑controlled trial in people with MS and neurogenic lower urinary tract dysfunction, treated with CBD (50 mg/day for 15 weeks) and planned outcomes for urinary dysfunction and unknown trial size. NCT05269628 is an active, randomized, double‑blind, three‑arm, placebo‑controlled trial in people with MS, planned CBD (Epidiolex® 100 mg twice daily) treatment of MS sleep disruption and pain, with an estimated enrollment of ~ 166. NCT05092191 is an active, double‑blinded, randomized, factorial, placebo‑controlled, parallel‑group trial in people with MS with estimated enrollment of ~ 250 split across four arms, with a CBD (200 mg/day) monotherapy arm with primary endpoints measuring MS spasticity and secondary outcomes measuring pain, sleep, physical function, quality of life and safety. We are unaware of MS clinical trials investigating non-psychotropic cannabinoids other than CBD.

#### HD

HD is a progressive, fully penetrant, autosomal dominant neurodegenerative disorder characterized by choreiform movement disorders, cognitive impairment, and psychiatric disturbances such as depression, irritability, and apathy (Walker 2007). HD manifests with selective neuronal loss and striatal atrophy primarily in the medium spiny neurons (MSNs) of the dorsal striatum, leading to impaired motor control, executive dysfunction, and emotional dysregulation as striato-cortical circuits degenerate (Morigaki and Goto 2017). HD is monogenic, caused by CAG trinucleotide repeat expansions (≥ 36—40 repeats) in exon 1 of the *HTT* gene, encoding mutant huntingtin (mHTT) protein with an expanded polyglutamine (polyQ) tract, where longer CAG repeats inversely correlate with age of onset (typically 30—50 years), and somatic CAG instability in brain tissue accelerates progression (Gatto et al., 2020).

HD pathology originates from intranuclear and cytoplasmic accumulation of mHTT aggregates (inclusion bodies) in neurons and glia, most prominently in striatum, cortex, and hypothalamic nuclei, which undergo prion-like seeding, transcriptional dysregulation, and proteostasis collapse (Tong et al., 2024). Mutant HTT impairs axonal transport, mitochondrial function, and synaptic integrity in striatal MSNs, while soluble polyQ-expanded fragments disrupt proteasomal and autophagic clearance, leading to excitotoxicity, caspase activation, and apoptosis in vulnerable indirect pathway neurons (Bergonzoni et al., 2021). mHTT also dysregulates BDNF signaling, depleting neurotrophic support from cortical afferents, and induces transcriptional repression of striatal genes essential for MSN identity and function (Gauthier et al., 2004).

Chronic microglia-mediated neuroinflammation is now recognized as a key amplifier of HD pathogenesis, transitioning from compensatory to maladaptive over disease course (Abedrabbo et al., 2025). Microglia express mHTT, which alters their transcriptional and functional phenotype in a cell-autonomous manner, where mHTT interacts with NF-κB signaling components, promoting constitutive NF-κB hyperactivation and enhanced transcription of pro-inflammatory cytokines (Crotti et al., 2014). This drives a chronically “primed” state in which microglia respond to secondary stimuli such as neuronal debris, misfolded proteins, and metabolic stress with exaggerated cytokine release. In this model, neuronal mHTT aggregates and other ligands from stressed and dying MSNs likely activate microglial TLRs which drives NLRP3 inflammasome maturation and release of IL-1β and IL-18, further escalating neuroinflammatory signaling (Saba et al., 2022). PET imaging reveals microglial activation detectable in premanifest HD up to 15 years before onset in striatum and cortex (Tai et al., 2007). Postmortem analyses confirm CD68 +/Iba1 + activated microglia colocalizing with mHTT inclusions and neuronal loss, underscoring neuroinflammation's role in progression beyond primary mHTT toxicity (Wilton et al., 2023).

In contrast to the other neurodegenerative diseases discussed so far, CBG is the primary non-psychotropic phytocannabinoid with preclinical HD evidence demonstrating a direct anti-inflammatory microglial effect. In a 3-nitropropionate (3-NP) mouse model, CBG (10 mg/kg, i.p.) improved motor deficits and preserved striatal neurons, while significantly attenuating reactive Iba1 + microgliosis and reducing the upregulation of TNF‑α, iNOS, and IL-4 induced by 3-NP (Valdeolivas et al., 2015). In the same study, using a R6/2 transgenic HD mouse containing an expanded polyglutamine mHTT, CBG (10 mg/kg, i.p.) modestly improved motor performance and partially normalized expression of several disease-associated genes including BDNF, IGF-1, PPARγ, CBD and TNF‑α, although microglial-specific assays were not tested in this model. CBD (5 mg/kg, i.p.) was also neuroprotective in the striatum of 3-NP lesioned mice where it increased substance P, proenkephalin, neuronal-specific enolase and SOD levels, and notably functioned independent of CB1, TRPV1, and A2AR signaling (Sagredo et al., 2007); however, this study also did not assay microglial-specific effects.

In 1991, a double‑blind, randomized, placebo‑controlled, crossover trial (*n* = 15 HD patients) tested CBD at 10 mg/kg/day (~ 700 mg/day average) for 6 weeks vs placebo, but found no significant improvement in chorea or motor, cognitive, and psychiatric HD symptoms (Consroe et al., 1991). We are unaware of any other HD clinical trials testing non-psychotropic phytocannabinoids.

## Conclusion

Non-psychotropic cannabinoids from cannabis represent a pharmacologically diverse class of compounds with substantial promise for mitigating microglial-mediated neuroinflammation across neurodegenerative diseases. Recent plant breeding efforts and new genetic understanding of cannabinoid biosynthesis have provided novel non-psychotropic cannabis chemotypes and cultivars, particularly in Type 3 and 4 hemp and varin-rich and CBC(A)-rich subtypes, for which cost-effective techniques can extract cannabinoids of interest. These plants will be important for fueling interest in and providing cannabinoids in addition to CBD to test against microglial-mediated inflammatory mechanisms. Detailed understanding of the genetics that control cannabinoid production in cannabis is critical because it links specific alleles and loci to predictable chemotypes and cannabinoid contents, enabling breeding and commercialization of therapeutically-targeted cultivars across the full spectrum of major and minor phytocannabinoids. Further, because psychotropic THC content is commonly used to delineate and define legal cannabis products such as hemp, knowledge of cannabinoid biosynthesis is crucial for developing cultivars which adhere to regulated markets. For example, non-specific THCA production by CBDAS or hypofunctional CBDAS or THCAS alleles could lead to THC(A) concentrations higher than allowed limits in many regulatory jurisdictions.

Across the rapidly expanding phytocannabinoid literature, one theme is increasingly clear: although CBD remains the dominant non-psychotropic cannabinoid investigated across microglial biology and neurodegeneration, our mechanistic understanding of the broader phytocannabinoid landscape is still profoundly incomplete. Outside of CBD, and to a far more limited degree CBG in HD models, virtually no preclinical work has delineated how other non-psychotropic phytocannabinoids modulate microglial signaling, phenotype switching, metabolic state, phagocytic programs, or cytokine output. Even for CBD, microglia-specific mechanisms remain insufficiently directly demonstrated and isolated from effects on astrocytes, neurons, and heterologous cell models. For example, CBD is the only non-psychotropic phytocannabinoid that has been shown to have direct microglial MOAs through targets discussed in this review, and only through a handful of receptors: CB2, TRPV1, TRPV2, and PPARγ. Additionally, no non-psychotropic phytocannabinoids have been shown to desensitize target receptors directly in microglia; these effects have been inferred largely from studies expressing channels in heterologous cells. This absence of mechanistic resolution is particularly evident for cannabinoids that are emerging as strong novel candidates for microglia-targeted therapeutics. CBG, CBC, the varinic analogues, and the acidic cannabinoids all show pharmacological properties that should, in principle, map onto microglial pathways relevant to neuroinflammation. For example, CBDA (10 mg/kg, i.p.) was most effective at reducing microglial reactivity in a TDP-43 transgenic mouse ALS model that also screened CBD, CBDV, and THCV as potential therapeutics (García-Toscano et al., 2025), although microglial MOAs were not tested. Future research must prioritize microglia-specific assays targeting progressive disease stages where chronic microglial activation predominates.

Clinically, the void beyond CBD is stark; no trials have evaluated CBG, CBC, or varins/acids in neurodegenerative cohorts, despite preclinical rationale. Based on completed clinical trials of CBD, it may be a promising therapeutic in AD, but does not appear particularly effective in PD, MS, and HD. However, these trials have had relatively small CBD treatment arms and may be underpowered to determine therapeutic significance. Epidiolex® brand CBD is currently the only FDA-approved non-psychotropic phytocannabinoid medication in the United States and is only approved for the treatment of seizures associated with Lennox-Gastaut syndrome, Dravet syndrome, and tuberous sclerosis complex in patients one year of age and older. Notably, Epidiolex® is a highly purified, cannabis plant-derived medication. Newer cannabis chemotypes rich in non-psychotropic cannabinoids other than CBD(A) therefore provide a toolkit to identify and test potentially promising new therapies for neurodegenerative diseases.

Future inflammation studies in primary microglial cultures and microglial cell lines like BV-2, could in theory test all the non-psychotropic phytocannabinoids in parallel to definitively isolate the most important molecular targets and signaling pathways, delineate unique and overlapping MOAs between cannabinoids, and identify the most promising cannabinoids for further pre-clinical models and clinical trials. However, the current number of potential phytocannabinoid MOAs is large and more continue to be discovered, making piecemeal dissection of molecular signaling pathways likely. For example in BV-2 microglial cells, CBDV restrained LPS-induced activation of inflammation by binding and reducing the stability of TLR4 co-receptor myeloid differentiation factor 2 (MD2) (Wang et al., 2022b). Additionally, non-psychotropic phytocannabinoids (primarily CBD) have been shown to directly modulate voltage-gated sodium, potassium, and calcium channels, other GPCRs, and receptor tyrosine kinases (Bih et al., 2015), many of which are expressed in microglia and could play functional roles in inflammation. Of particular importance, non-psychotropic phytocannabinoids may not realistically reach therapeutic micromolar concentrations in brain and spinal cord microglial niches that are necessary for many effects observed *in vitro*, due to issues with oral bioavailability, PK, and BBB. If this is the case, it would limit the known plausible interaction spectrum of non-psychotropic phytocannabinoids with molecular targets to those in the nanomolar range (e.g. interactions marked with an asterisk on Fig. 2): CBD at CB2, GPR55, A2AR, D2DR, TRPA1 and TRPM8; CBG at α_2_-AR and TRPM8; CBC at TRPA1; THCV at CB2; and THCA at TRPM8. This would considerably simplify testing a full MOA panel for future microglial studies.

The unique, if complex, polypharmacology of non-psychotropic phytocannabinoids sets them apart from selective compounds as novel therapeutics for microglial-mediated neurodegeneration. These cannabinoids exhibit distinct, yet sometimes complementary pharmacological profiles at microglial targets, offering a compelling rationale for mixed-cannabinoid formulations to achieve synergistic or additive therapeutic effects in neuroinflammation. For instance, CBG's high-potency α_2_-AR agonism could, in theory, synergize with CBD's high-potency, anti-inflammatory effects at other receptors. Furthermore, the advent of iPSC models of neurodegeneration could facilitate investigation of cannabinoids or combinations in high-risk patient-derived samples, with future implications for personalized medicine. Finally, most non-psychotropic phytocannabinoids are not scheduled, restricted compounds like THC in the U.S. and several other major regulatory jurisdictions, and novel cannabis genetics should facilitate future pre-clinical and clinical studies with minimal regulatory barriers.

## Data Availability

No datasets were generated or analysed during the current study.

## Abbreviations

2-AG: 2-Arachidonoylglycerol
3-NP: 3-Nitropropionate
A2AR: Adenosine A2A receptor
AA: Arachidonic acid
Aβ: Amyloid-β
AC: Adenylyl cyclase
AD: Alzheimer’s disease
AE: Autoimmune encephalomyelitis
AEA: Anandamide
ALS: Amyotrophic lateral sclerosis
AMPK: AMP-activated protein kinase
APP: Amyloid precursor protein
Arg-1: Arginase-1
ASC: Apoptosis-associated speck-like protein containing a CARD
ATP: Adenosine triphosphate
BACE-1: β-Site APP-cleaving enzyme 1
BDNF: Brain-derived neurotrophic factor
BDS: Botanical drug substance
BPSD: Behavioral and psychological symptoms of dementia
BuChE: Butyrylcholinesterase
cAMP: Cyclic adenosine monophosphate
CB1: Cannabinoid receptor 1
CB2: Cannabinoid receptor 2
CBC: Cannabichromene
CBCA: Cannabichromenic Acid
CBCAS: Cannabichromenic Acid Synthase
CBCV: Cannabichromevarin
CBCVA: Cannabichromevarinic Acid
CBD: Cannabidiol
CBDA: Cannabidiolic Acid
CBDAS: Cannabidiolic Acid Synthase
CBDV: Cannabidivarin
CBDVA: Cannabidivarinic Acid
CBG: Cannabigerol
CBGA: Cannabigerolic Acid
CBGAS: Cannabigerolic Acid Synthase
CBGV: Cannabigerovarin
CBGVA: Cannabigerovarinic Acid
CCL: C-C motif chemokine ligand
CNS: Central Nervous System
COX: Cyclooxygenase
CXCL: C-X-C motif chemokine ligand
DAGL: Diacylglycerol lipase
DAMGO: [D-Ala2, N-MePhe4, Gly-ol]encephalin
DAMP: Damage-associated molecular pattern
DEA: Drug Enforcement Administration
EAE: Experimental autoimmune encephalomyelitis
ECS: Endocannabinoid System
EET: Epoxyeicosatrienoic Acid
ERK1/2: Extracellular signal-regulated kinase ½
FAAH: Fatty Acid Amide Hydrolase
FDA: Food and Drug Administration
GABA: γ-Aminobutyric acid
GDNF: Glial cell line–derived neurotrophic factor
GPCR: G protein-coupled receptor
GPP: Geranyldiphosphate
GPR12: G protein-coupled receptor 12
GPR3: G protein-coupled receptor 3
GPR55: G protein-coupled receptor 55
GSK3β: Glycogen synthase kinase 3β
H4CBD: Hexahydrocannabidiol
HD: Huntington’s Disease
HHC: Hexahydrocannabinol
HIV-1: Human immunodeficiency virus type 1
HO-1: Heme oxygenase-1
HTT: Huntingtin
Iba1: Ionized calcium-binding adaptor molecule 1
IFNγ: Interferon-γ
IGF-1: Insulin-like growth factor 1
IL: Interleukin
IL-1β: Interleukin-1 beta
IL-4: Interleukin-4
IL-6: Interleukin-6
IL-8: Interleukin-8
IL-10: Interleukin-10
IL-12p70: Interleukin-12 p70
IL-18: Interleukin-18
IL-23: Interleukin-23
iNOS: Inducible nitric oxide synthase
IP3R: Inositol 1,4,5-trisphosphate receptor
IPSC: Induced pluripotent stem cell
i.c.v.: Intracerebroventricular
i.g.: Intragastric
i.p.: Intraperitoneal
IVH: Intraventricular Hemorrhage
JNK: C-Jun N-terminal kinase
L-DOPA: L-3,4-dihydroxyphenylalanine
LPS: Lipopolysaccharide
LRRK2: Leucine-rich repeat kinase 2
MAGL: Monoacylglycerol lipase
MAPK: Mitogen-activated protein kinase
MD2: Myeloid differentiation factor 2
mHTT: Mutant huntingtin
MKP-1: Mitogen-activated protein kinase phosphatase-1
MOG: Myelin oligodendrocyte glycoprotein
MOR: μ-Opioid receptor
MS: Multiple Sclerosis
MSN: Medium Spiny Neuron
MTDL: Multi-target-directed ligand
NAPE-PLD: N-acyl-phosphatidylethanolamine-specific phospholipase D
NAWM: Normal-appearing white matter
NF-κB: Nuclear factor kappa-light-chain-enhancer of activated B cells
NIH: National Institutes of Health
NK: Natural killer
NLRP3: NLR family pyrin domain containing 3
NO: Nitric Oxide
NOS: Nitric Oxide Synthase
Nrf2: Nuclear factor erythroid 2–related factor 2
p.o.: Oral
PD: Parkinson’s Disease
PET: Positron Emission Tomography
PIP2: Phosphatidylinositol 4,5-bisphosphate
PK: Pharmacokinetic
PKA: Protein kinase A
PKC: Protein kinase C
PP2A: Protein phosphatase 2A
PPAR: Peroxisome Proliferator-activated Receptor
PPMS: Primary Progressive Multiple Sclerosis
PSEN: Presenilin
PTSD: Post-traumatic Stress Disorder
RAGE: Receptor for Advanced Glycation Endproducts
RCT: Randomized Controlled Trial
RNS: Reactive Nitrogen Species
ROS: Reactive Oxygen Species
RRMS: Relapsing-remitting multiple sclerosis
s.c.: Subcutaneous
SNc: Substantia nigra pars compacta
SPMS: Secondary progressive multiple sclerosis
STAT: Signal transducer and activator of transcription
SYK: Spleen tyrosine kinase
TDP-43: TAR DNA-binding protein 43
TGF-β: Transforming growth factor-β
TH: Tyrosine hydroxylase
THC: Δ⁹-Tetrahydrocannabinol
THCA: Δ⁹-Tetrahydrocannabinolic acid
THCAS: Tetrahydrocannabinolic acid synthase
THCP: Δ⁹-Tetrahydrocannabiphorol
THCV: Δ⁹-Tetrahydrocannabivarin
THCVA: Δ⁹-Tetrahydrocannabivarinic acid
TLR: Toll-like receptor
TNF-α: Tumor necrosis factor-alpha
TREM2: Triggering Receptor Expressed on Myeloid cells 2
TRP: Transient Receptor Potential
TRPA: Transient Receptor Potential Ankyrin
TRPC: Transient Receptor Potential Canonical
TRPV: Transient Receptor Potential Vanilloid
TRPM: Transient Receptor Potential Melastatin
TSPO: Translocator Protein
ULK1: Unc-51-like autophagy activating kinase 1
UPDRS: Unified Parkinson’s Disease Rating Scale
w/w: Weight/weight
